# Maternal smoking and high BMI disrupt thyroid gland development

**DOI:** 10.1186/s12916-018-1183-7

**Published:** 2018-10-23

**Authors:** Panagiotis Filis, Sabine Hombach-Klonisch, Pierre Ayotte, Nalin Nagrath, Ugo Soffientini, Thomas Klonisch, Peter O’Shaughnessy, Paul A. Fowler

**Affiliations:** 1 Institute of Medical Sciences, School of Medicine, Medical Sciences and Nutrition, University of Aberdeen, Foresterhill, Aberdeen, AB25 2ZD UK; 2 Institute of Biodiversity, Animal Health & Comparative Medicine (IBAHCM), College of Medical, Veterinary & Life Sciences, University of Glasgow, Garscube Campus, Bearsden Rd, Glasgow, G61 1QH UK; 3 Department of Human Anatomy and Cell Science, Rady College of Medicine, Faculty of Health Sciences, University of Manitoba, Winnipeg, MB Canada; 4 Centre de toxicologie, Institut National de Santé Publique du Québec, Quebec, QC G1V 5B3 Canada

**Keywords:** Fetus, Human, Thyroid, Maternal smoking, Maternal obesity, Thyroid hormones, Development

## Abstract

**Background:**

Maternal lifestyle factors, including smoking and increased body weight, increase risks of adult diseases such as metabolic syndrome and infertility. The fetal thyroid gland is essential for the control of fetal metabolic rate, cardiac output, and brain development. Altered fetal thyroid function may contribute to increased disease onset later in life. Here, we investigated the impact of maternal smoking and high maternal weight on human fetal thyroid function during the second trimester.

**Methods:**

Thyroid glands and plasma were collected from fetuses electively terminated in the second trimester (normally progressing pregnancies). Plasma total triiodothyronine (T3) and total thyroxine (T4) were measured by solid-phase extraction-liquid chromatography-tandem mass spectrometry. Fetal plasma thyroid-stimulating hormone (TSH) levels were measured using a multiplex assay for human pituitary hormones. Histology and immunolocalization of thyroid developmental markers were examined in thyroid sections. Transcript levels of developmental, functional, apoptotic, and detoxification markers were measured by real-time PCR. Statistical analyses were performed using multivariate linear regression models with fetal age, sex, and maternal smoking or maternal body mass index (BMI) as covariates.

**Results:**

Maternal smoking was associated with significant changes in fetal plasma T4 and TSH levels during the second trimester. Smoke-exposed thyroids had reduced thyroid *GATA6* and *NKX2-1* transcript levels and altered developmental trajectories for *ESR2* and *AHR* transcript levels. Maternal BMI > 25 was associated with increased fetal thyroid weight, increased plasma TSH levels, and abnormal thyroid histology in female fetuses. Normal developmental changes in *AHR* and *ESR1* transcript expression were also abolished in fetal thyroids from mothers with BMI > 25.

**Conclusions:**

For the first time, we show that maternal smoking and high maternal BMI are associated with disturbed fetal thyroid gland development and endocrine function in a sex-specific manner during the second trimester. These findings suggest that predisposition to post-natal disease is mediated, in part, by altered fetal thyroid gland development.

**Electronic supplementary material:**

The online version of this article (10.1186/s12916-018-1183-7) contains supplementary material, which is available to authorized users.

## Background

The thyroid gland is essential for normal human fetal development and is the source of the hormones triiodothyronine (T3) and thyroxine (T4). The thyroid primarily secretes T4, which is converted to the more active T3 by tissue-specific deiodinase enzymes. Thyroid hormones control metabolic rate, cardiac output, and brain function and are critical for normal fetal brain development and growth [1]. During the first trimester, T3 and T4 in the human fetal circulation are of maternal origin, but from the start of the second trimester, the developing fetal thyroid increasingly contributes to T3 and T4 levels in the fetal circulation [2, 3].

The “fetal origins of adult disease” hypothesis states that a diverse array of diseases in the adult, ranging from metabolic syndrome and obesity to cardiovascular or behavioral disorders, originates during fetal life [4]. In pregnant women, lifestyle factors such as high body weight, medication use, drug abuse, stress, alcohol consumption, and smoking are known to have an impact on the long-term health outcomes of the offspring. Typically, 31% of women aged under 30 years smoke when first pregnant and fewer than 4% of pregnant women stop smoking during pregnancy [5]. Maternal smoking during pregnancy has immediate adverse effects including pre-term delivery and stillbirth and has been linked to metabolic syndrome [6, 7], low birth weight, reduced fertility [8, 9], increased miscarriage rates [10], and psychosomatic problems in the offspring [11]. High maternal body mass index (BMI) is the most prevalent adverse lifestyle factor in women of reproductive age, with 64% of women overweight and 35% obese in the USA and UK [12, 13]. Elevated BMI during pregnancy predisposes the offspring to the highest mortality-inducing diseases worldwide such as premature death from cardiovascular disease [14], obesity and metabolic syndrome [15], and respiratory complications [16] and to other conditions such as autism [17].

The endocrine functions of the thyroid gland are critical in controlling metabolic rate and brain development, and altered thyroid functions during fetal life may alter the predisposition of the offspring to childhood and adult disease. Despite this, little is known about the effects of adverse maternal lifestyles on fetal thyroid gland development. It has been shown that neonates exposed to cigarette smoke in utero have enlarged thyroid glands [18] while maternal smoking increases free T3 levels in maternal serum and reduces neonate cord serum thyroid-stimulating hormone (TSH) levels [19]. Similarly, increased maternal and neonate weight correlate with increased free T3 levels in neonatal cord serum [20]. It is currently unclear, however, at what stage of pregnancy these effects of maternal smoking and/or increased maternal weight begin to affect the development and/or function of the fetal thyroid gland in utero. Knowing the developmental windows of sensitivity in the fetus, however, may help lead to informed lifestyle changes by prospective parents and could initiate the development of early preventive and/or ameliorative strategies for children at risk.

In the present study, we have examined human fetal thyroid development during the second trimester using tissues from normally progressing electively terminated pregnancies. In a step-change in the field, we have measured circulating fetal plasma levels of total T3, T4, and TSH, as well as levels of the thyroid hormone-binding proteins albumin (ALB), transthyretin (TTR), and thyroxine-binding globulin (TBG). In addition, we have examined fetal thyroid morphology and quantified the transcript levels of genes related to thyroid development and function. Our results indicate that maternal smoking and high maternal weight trigger complex and sex-specific effects during the second trimester of pregnancy which affect thyroid gland morphology and endocrine functions.

## Methods

### Sample collection and processing

The collection of fetal material was approved by the NHS Grampian Research Ethics Committee (REC 04/S0802/21). Women seeking elective, medical terminations of pregnancy were recruited with written informed consent. Only normally progressing pregnancies from women over 16 years of age and between 11 and 21 weeks of gestation were collected. Fetal plasma samples were collected by ex vivo cardiac puncture, and plasma was separated by centrifugation in heparin tubes and snap-frozen. Fetal thyroids were dissected, weighted, and snap-frozen (right lobe) or fixed in 4% formalin overnight and embedded in paraffin (left lobe). All frozen tissues were stored at − 80 °C. Fetal sex was determined by gonadal histology. Maternal smoke exposure was confirmed by cotinine measurements as previously described, and samples were categorized as control (C) or smoke-exposed (SE) [21]. Maternal BMI information was collected as part of the patient consent procedure, and women were categorized as normal weight (17.5 < BMI < 25) or overweight/obese (BMI ≥ 25). Different subsets from a total of 163 fetal plasma and fetal thyroid samples were used for (i) thyroid weights, (ii) circulating hormonal measurements, (iii) circulating thyroid hormone binding proteins, (iv) fetal thyroid morphological examination, (v) immunohistochemistry, and (vi) transcript measurements. Detailed maternal and fetal characteristics, as well as sample numbers in each category, are provided in Additional file 1: Figure S1. Only blood samples and thyroid tissues obtained within 30–60 min from the time of delivery were used in this study. The weights of 94 fetal thyroid glands were measured (*smoke exposure study*: 26 control females, 25 control males, 14 smoke-exposed females, and 29 smoke-exposed males; *maternal BMI study*: 24 normal weight females, 26 normal weight males, 16 overweight/obese females, and 27 overweight/obese males, Additional file 1: Figure S1A). Mean fetal age was not different among smoking or BMI groups in either sex, and mean BMI values did not differ by smoke exposure or sex among the fetal samples used for thyroid weight (Additional file 1: Figure S1A).

### Plasma fetal hormonal measurements

Total T3, total T4, and TSH were measured in a total of 60 fetal plasma samples from fetuses ranging between 12 and 20 weeks of gestation (Additional file 1: Figure S1B). Mean fetal age was not different among smoking or BMI groups in either sex, and mean BMI values did not differ by smoke exposure or sex among the fetal samples used for hormonal measurements (Additional file 1: Figure S1B). Total thyroid hormones were measured using a solid-phase extraction-liquid chromatography-tandem mass spectrometry method [22]. To 10-μL aliquots of fetal plasma, 2 mL of a 4% H_3_PO_4_ aqueous solution, 250 μL of a 60:40 methanol:water solution, and 50 μL of a 10 ppb solution of isotopically labeled [^13^C_6_]-T4 and [^13^C_6_]-T3 were added. Each sample was vortexed for 30 s and loaded on a SilicaPrepX SCX 60 mg/3 mL extraction cartridge preconditioned by adding 1 mL of methanol and 1 mL of the 4% H_3_PO_4_ aqueous solution. Cartridges were rinsed with 2 mL of water followed by 1 mL of methanol, and the compounds eluted with 1 mL of a methanol:ammonium hydroxide (95:5) solution. Solvents were evaporated to dryness in a Zymark Turbo Vap evaporator and the samples taken up in 500 μL of deionized (DI) water:methanol (50:50) containing 0.15% H_3_PO_4_. After vortex mixing, 250 μL of the resulting solution was transferred to a polypropylene tube for subsequent analysis by LC-MS/MS. A Xevo TQ-S electrospray ionization-triple quadrupole mass spectrometer coupled with an Acquity UPLC unit (UPLC-ESI-MS/MS, Waters, Manchester, U.K.) was used for thyroid hormone quantification. A 10-μL aliquot of the sample was injected into an ACE EXCEL C18-Ar column 50 × 2.1 mm, 2 μm. The flow rate was set to 0.4 mL/min and the column temperature to 30 °C. Initial conditions were 70% mobile phase A (0.1% acetic acid in DI-water) and 30% mobile phase B (methanol). The gradient went from 30% phase B to 80% within 4 min, followed by a wash for 30 s at 95% phase B and a return to initial conditions during the next 30 s. Thyroid hormones were monitored in positive ionization mode by MRM (multiple reaction monitoring). Selected transitions [M + H]+ were m/z 778 → m/z 732 (quantification) and m/z 778 → m/z 605 (qualifier) for T4 and m/z 652 → m/z 606 (quantification) and m/z 652 → m/z 479 (qualifier) for T3. Selected transitions [M + H]+ for isotopically labeled THs were m/z 784 → m/z 738 and m/z 784 → m/z 611 (qualifier) for [^13^C_6_]-T4 and m/z 658 → m/z 612 and m/z 658 → m/z 485 (qualifier) for [^13^C_6_]-T3. Standard curve ranged from 2.5 to 500 ng/mL for T4 and 0.25 to 50 ng/mL for T3. *R*^2^ was greater than 0.995 for all standards, as determined by TargetLynx (Version 4.1, Waters). To assess the accuracy of T3 and T4 determination, two quality controls (QC) from Bio-Rad [Lyphocheck® Assayed Chemistry Control lot #14440: level 1—14,441 (T4 = 71 ng/mL) and level 2—14,442 (T4 = 208 ng/mL)] were included in the analytical batch (*N* = 5 for both QCs). The accuracy (%bias) was − 2.8% for QC 14441 and − 9.7% for QC 14442, whereas the precision (%CV) was 3.0% for QC 14441 and 2.0% for QC 14442. No T3 concentrations are provided for these standards, but mean T3 concentrations of 0.87 ng/mL and 3.51 ng/mL were measured in QC 14441 and QC 14442, respectively. The precision of T3 determination was 7.2% for QC 14441 and 1.9% for QC 14442.

Plasma TSH levels were measured (in 25 μl/fetus) using a single Milliplex® MAP Human Pituitary Magnetic Bead Panel 1 kit (ACTH, GH, TSH, CNTF, AGRP: Millipore Limited, Watford, UK) and analyzed using a BioPlex200 system (Bio-Rad Laboratories Ltd., Hemel Hempstead, UK). Intra- and inter-assay coefficients of variation were < 10% and < 15%, respectively, sensitivity was 0.02 μIU/mL, and cross-reactivity was negligible.

### Measurements of circulating fetal thyroid hormone-binding proteins

Fetal plasma proteins were measured in a total of 80 fetal plasma samples ranging from 12 to 20 weeks of gestation (Additional file 1: Figure S1C). Mean fetal age was not different among smoking or BMI groups in either sex, and mean BMI values did not differ by smoke exposure or sex among the fetal samples used for circulating protein measurements (Additional file 1: Figure S1C). Ten micrograms of fetal plasma proteins were diluted to a final volume of 100 μL in 50 mM NH4HCO3 (BioUltra grade, Sigma Aldrich). Proteins were digested in solution according to the PRIME-XS protocol (http://www.primexs.eu/protocols/Public-Documents/04%2D%2D-Protocols/PRIME-XS-Protocol-NPC-In-Solution-Digestion.pdf/). Briefly, proteins were reduced in 2 mM dithiothreitol (Sigma Aldrich, > 99%) for 25 min at 60 °C and S-xalkylated in 4 mM iodoacetamide (Sigma Aldrich, > 99%) for 30 min at 25 °C in the dark, then digested by sequencing-grade modified trypsin (Promega, Southampton, UK, cat.no. V5111) at a 1:10 ratio of trypsin:protein overnight at 37 °C. The reaction was stopped by freezing at − 80 °C. Samples were then thawed, dried by vacuum centrifugation (SpeedVac Plus SC110A, Savant), and dissolved in 10 μL 2% acetonitrile/0.1% formic acid. The equivalent of 2 μg of peptides (assuming no losses) were analyzed by liquid chromatography-tandem mass spectrometry (LC-MS/MS). The LC-MS system included a Thermo Scientific Dionex UltiMate 3000 RSLC nano-LC configured for pre-concentration onto a nano-column, coupled to a Q Exactive Plus hybrid quadrupole-Orbitrap mass spectrometer fitted with an EASY-Spray nano-ESI source (Thermo Scientific). Peptide samples were injected onto a C18 PepMap 100 pre-column (300 μm i.d. × 5 mm) in loading pump solvent (2% acetonitrile, 0.1% formic acid) at a flow rate of 10 μL/min for 5 min. The pre-column was then reverse-flushed to the analytical column (PepMap RSLC C18; 50 μm i.d. × 15 cm; Nano pump solvent A: 0.1% formic acid, Nano pump solvent B: 80% acetonitrile, 0.1% formic acid) at 0.3 μL/min using the nano-pump. Peptides were separated using a gradient of acetonitrile (LC gradient: 3–10% solvent B in 5 min, 10–40% solvent B in 30 min, 40–80% solvent B in 5 min, hold at 80% solvent B for 8 min, 80–3% solvent B in 1 min, hold at 3% solvent B for 15 min) while MS/MS data were acquired by the Q Exactive in data-dependent mode (Top10 method). Parameters for the full scan/data-dependent MS2 (Top10) method were as follows: full scan range 375–1750 m/z, resolution 70,000, AGC target 3e6, and maximum IT 50 ms; MS2 scan resolution 17,500, AGC target 5e4, maximum IT 100 ms, loop count 10, isolation window 1.6 m/z, NCE 26, underfill ratio 4%, charge states 2–5 included, peptide match preferred, exclude isotopes on, and dynamic exclusion 40 s.

Raw mass spectrophotometric output files were processed by MaxQuant (v 1.5.3.30) [23]. MaxQuant runs were performed under the default parameters except that trypsin was set as the digestion enzyme. All peptide matching searches were performed against a FASTA file of the human proteome (20,437 canonical and isoform reviewed protein sequences, downloaded from Uniprot on the 6th of July 2018). Protein intensities across samples were normalized using the maxLFQ algorithm [24]. The normalized protein intensities for ALB, TTR, and TBG were extracted from the results file for the downstream statistical analysis.

### Fetal thyroid histology and immunohistochemistry

Histological sections from 83 fetal thyroid glands ranging from 11 to 20 weeks of gestation (Additional file 1: Figure S1D) were stained with hematoxylin and eosin (H&E) prior to morphological assessment. Histological sections from 55 fetal thyroid glands, ranging from 11 to 20 weeks of gestation (Additional file 1: Figure S1E), were immunohistochemically stained for paired box 8 (PAX8), forkhead box A2 (FOXA2), calcitonin, Na^+^-I^−^ Symporter (NIS, gene name: *SLC5A5*), and thyroid transcription factor 1 (TTF1, gene name: *NKX2-1*). Mean fetal age was not different among smoking or BMI groups in either sex, and mean BMI values did not differ by smoke exposure or sex among the fetal samples used for H&E thyroid morphology scoring, but in the fetal samples used for immunohistochemical scoring, smoke-exposed females had higher mean maternal BMI (Additional file 1: Figure S1D-E). For each thyroid specimen, two sections were immunostained with the respective antibodies. Sections were dewaxed and rehydrated in Tris-based saline containing 0.05% Tween-20 (TBST), and endogenous peroxidase was inactivated with 3% H_2_O_2_ in methanol for 20 min at room temperature (RT). Heat-antigen retrieval was achieved with citrate buffer (pH 6.0) for 20 min, and non-specific binding sites were saturated with TBST containing 5% goat normal serum. Incubations were done overnight at 4 °C in blocking buffer (NIS and TTF1) and for 1 h at RT (PAX8). Horse-radish peroxidase-conjugated anti-mouse or anti-rabbit secondary antibodies (both Jackson Immunoresearch, West Grove, PA, USA) were used at 1:200 for 1 h at RT. Antibody information is provided in Additional file 2: Table S1. Specific binding was visualized using the 3,3′-diaminobenzidine tetrahydrochloride (DAB) substrate (Pierce Biotechnology, Rockford, IL). Nuclei were counterstained with hematoxylin. Slides were imaged using a Zeiss Axio Imager M.2 microscope with color camera and Zeiss Zen software (Zeiss, Jena, Germany). For PAX8, calcitonin, and NIS, immunostained sections were scored according to the number of positively stained cells ranging from −, no staining; (+), few cells; +, more cells; and ++, strong many cells. FOXA2-immunostained sections were scored according to staining localization ranging from −, no staining; (+), cytoplasmic staining; +, cytoplasmic and nuclear staining; and ++, nuclear staining. Thyroid morphology was examined on two 5-μm-thick sections which were sectioned 50 μm apart from each other. Thyroid sections were assessed at the glandular margin to avoid comparing central with peripheral parts of the thyroid gland and were independently examined by two observers blinded to the information on fetal age, sex, smoke exposure, and maternal BMI. The H&E morphology was observed throughout the spectrum of 11–21 gestational weeks.

### Transcript measurements

RNA was extracted from 78 whole fetal thyroid tissues ranging from 11 to 20 weeks of gestation (Additional file 1: Figure S1F) using Qiagen All-prep kits (#80004, Qiagen, Manchester, UK) and then reverse transcribed as described previously [25]. In order to identify the most appropriate housekeeping gene (HKG) for normalization of fetal thyroid mRNA transcript levels, the expression of five HKGs (*SDHA*, *SFRS4*, *B2M*, *TBP*, and *PMM1*) [25] was assessed using GeNorm and Normfinder algorithms [26, 27]. *SDHA* was found to be the most stable HKG using Normfinder while GeNorm identified a combination of *SDHA* and *SFRS4*. Transcript measurements were subsequently normalized against *SDHA* in all studies reported here. A list of all the primers used is provided in Additional file 3: Table S2. Mean fetal age for either sex was similar in the smoke exposure and maternal BMI groups. In the fetal samples used for thyroid transcript measurements, mean BMI values were similar between sexes and in the smoke exposure group (Additional file 1: Figure S1F).

### Statistical analyses

All statistical analyses were performed in R statistical software (V3.4.0). Thyroid weight and hormonal, protein, and transcript measurements were analyzed using multivariate linear regression models; thyroid morphological scoring of H&E sections was compared among groups using multivariate generalized linear models for binomial data (mature vs immature); and immunohistochemical staining scores were analyzed using ordinal logistic regression. Covariates for all analyses were fetal age (continuous), fetal sex (categorical), maternal smoke exposure (categorical), and maternal BMI (categorical). Two major models were employed to examine the relationship between (i) fetal age, fetal sex, and maternal smoking and (ii) fetal age, fetal sex, and maternal BMI. Initially, the presence of three-way age-sex-smoke exposure (or maternal BMI) interaction was examined with the full model: *Y*~age*sex*smoking (or maternal BMI). For those cases where no statistical evidence for a three-way age-sex-smoking (or maternal BMI) interaction was found, the three-way interaction model was replaced with the model containing all two-way interaction: *Y*~age*sex +age*smoking (or maternal BMI) + sex*smoking (or maternal BMI). For those cases where no statistical evidence for two-way interactions was found, the simple one-way model was used: *Y*~age+sex+smoking (or maternal BMI). For those cases where significant interactions were detected (*P* < 0.05), the dataset was split by sex or smoke exposure (or maternal BMI) to examine the source of interactions and to simplify model interpretation. Correlations between TSH levels and fetal plasma cotinine levels (continuous), fetal T4 levels (continuous), and maternal BMI (continuous) were performed using TSH~cotinine, TSH~BMI, and TSH~T4 linear models, respectively (Pearson’s correlation). Associations between maternal BMI and TSH in the female fetuses were examined by stratifying females into control and smoke-exposed groups and then examining BMI associations using TSH~age+BMI(categorical) models for each smoke exposure group, ensuring absence of significant two-way interactions between age and BMI. For thyroid gland weight, hormonal measurements, protein, and transcript measurements, where multivariate linear regression was used, normal distribution of the residuals and homoscedasticity were visually assessed by quantile-quantile residual plots and scatter plot of residuals vs fitted model values, respectively. Data were log-transformed in those cases where model residuals departed from normality and/or showed heteroscedasticity. Statistical significance threshold was chosen at a *P* value of < 0.05. To ensure that maternal smoke exposure does not interact with maternal BMI, preliminary statistical models were used to ensure that there are no four-way (age*sex*smoking*maternal BMI), no three-way (age*smoking*maternal BMI and sex* smoking*maternal BMI), and no two-way interactions (smoking*maternal BMI) between smoke exposure and maternal BMI. Generated *P* values, effect sizes (where applicable), and interactions for the statistical tests performed are given in Additional file 3: Table S2, Additional file 4: Table S3 and Additional file 5: Table S4.

## Results

### The roles of fetal age and sex in the developing human fetal thyroid

Total T3 and T4 levels in the fetal circulation increased with age, the T3/T4 ratio decreased with fetal age, and the thyroid hormone-binding proteins ALB and TBG increased with increasing fetal age in both males and females regardless of smoke exposure or maternal BMI models (Additional file 6: Figure S2A, Table 1). TSH levels did not change with fetal age in males and females not exposed to maternal smoking (Table 1). Male fetuses had higher T3 levels and a T3/T4 ratio compared to females (Table 1, Additional file 6: Figure S2A). There was no difference in thyroid gland weight between males and females, but male thyroids tended to show more immature thyroid gland morphology compared to female thyroids and this approached statistical significance (*P* = 0.057, Additional file 4: Table S3).

**Table 1:** Significance (*P* values) of associations between fetal age, sex, smoke exposure, high maternal BMI, and their interactions (two-way and three-way analyses), in circulating plasma hormones (T3, T4, TSH) and plasma thyroid hormone-binding proteins (ALB, TTR, TBG)

Interactions:	3-way	2-way				1-way		
Smoke effect	Age-sex-SE	Dataset splits	Age-sex	Age-SE	Sex-SE	Age	Sex	SE
T3 (nM)	0.38		0.55	0.56	0.85	*0.002* (*↑*)	*0.027* (*+ 1.4 fold* ♂)	0.67
T4 (nM)	0.098		0.65	*0.030*	0.89	N/A	N/A	*Interaction*
		Exposure split						
		C	0.19	N/A	N/A	*0.002* (*↑*)	0.84	N/A
		SE	0.38	N/A	N/A	*< 0.00001* (*↑*)	0.96	N/A
T3/T4 ratio	0.65		0.16	0.86	0.79	*0.0004* (*↓*)	*0.027* (*+ 1.4 fold* ♂)	0.72
`TSH (μIU/ml)	*0.003*	Sex split						
		**♂**	N/A	0.23	N/A	0.28	N/A	0.47
		**♀**	N/A	*0.003*	N/A	0.092 (↓C)	N/A	*Interaction*
		Exposure split						
		C	0.12	N/A	N/A	0.24	0.14	N/A
		SE	*0.008*	N/A	N/A	*0.017* (*↑♀*)	*Interaction*	N/A
ALB	0.63		0.16	0.58	0.1	*< 0.00001* (*↑*)	0.35	0.420
TTR	0.20		0.11	0.81	0.41	0.17	0.17	0.63
TBG	0.11		0.34	0.14	0.58	*0.0022* (*↑*)	0.89	0.13
BMI effect	Age-Sex-BMI		Age-sex	Age-BMI	Sex-BMI	Age	Sex	BMI
T3 (nM)	0.21		0.86	0.87	0.49	*0.002* (*↑*)	*0.021* (*+ 1.4 fold ♂*)	0.53
T4 (nM)	0.87		0.71	0.85	0.76	*< 0.0001* (*↑*)	0.74	0.49
T3/T4 ratio	0.28		0.89	0.99	0.36	*0.0003* (*↓*)	*0.013* (*+ 1.4 fold ♂*)	0.24
TSH (μIU/ml)	0.068		0.16	0.62	*0.005*	N/A	*Interaction*	*Interaction*
		Sex split						
		♂	N/A	0.14	N/A	0.25	N/A	0.27
		**♀**	N/A	0.26	N/A	0.23	N/A	*0.0071* (*+ 1.6 fold*)
ALB	0.74		0.15	0.48	0.72	*< 0.00001* (*↑*)	0.39	0.49
TTR	0.24		0.11	0.48	0.10	0.16	0.15	0.23
TBG	0.95		0.25	0.36	0.34	*0.0022* (*↑*)	0.88	0.13

Data were log-transformed where model residuals departed from normality and/or showed heteroscedasticity. Statistically significant differences (*P* < 0.05) are shown in italics. *C* controls, *SE* smoke-exposed, *N/A* not applicable

**Fig. 1:**
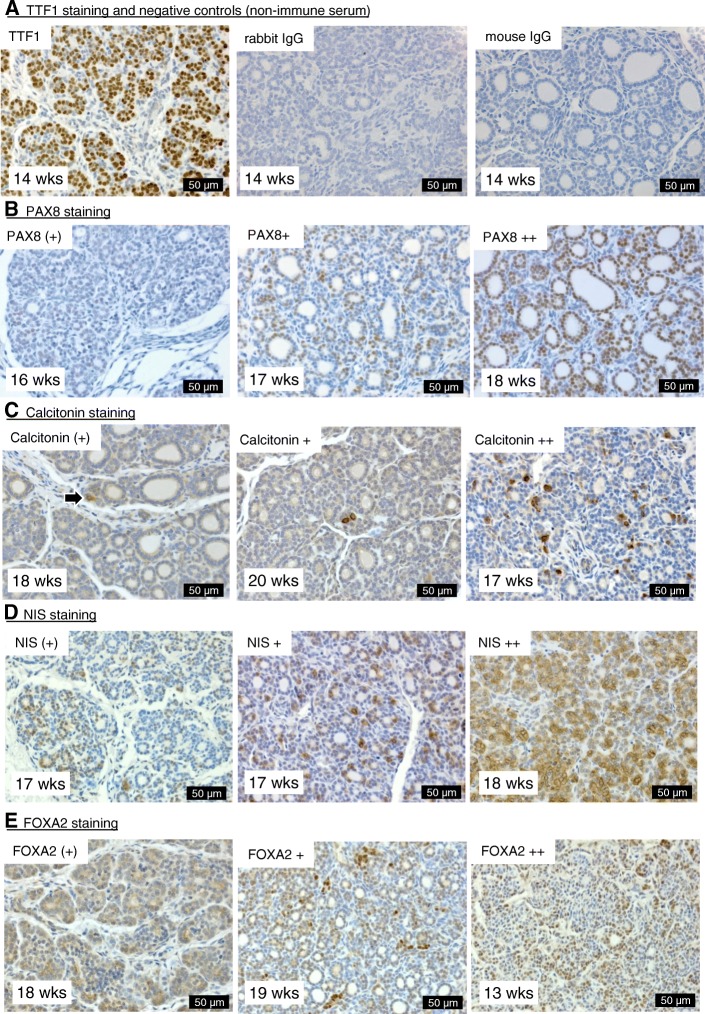
Immunohistochemical staining of human fetal thyroid sections. Representative images are shown for the immunostaining for TTF1, PAX8, calcitonin, NIS, and FOXA2 (**a**–**e**) including examples of the different scores ranging from weak (+) to strong ++. Negative controls that were not probed with primary antibodies are shown in panel **a**. Gestational ages (weeks) and scale bars (μm) are shown on the bottom left and right side of each image, respectively. Arrow in panel **c** indicates stained cell

Analysis of immunohistochemistry showed that TTF1 was invariably present in the nuclei of follicular cells of all sections examined while PAX8 staining ranged from absent to progressively present in more follicular cell nuclei (Fig. 1a, b). Calcitonin staining ranged from absent to increasingly present in parafollicular cells, and NIS staining ranged from absent to mostly cytoplasmic in a few follicular cells to more plasma membrane-localized in larger number of cells (Fig. 1c, d). FOXA2 staining ranged from absent to predominantly cytoplasmic, to cytoplasmic and nuclear, and predominantly nuclear (Fig. 1e). Irrespective of the statistical model used (age-sex-smoke exposure or age-sex-maternal BMI), PAX8 immunohistochemical scores remained constant in males but increased by gestation age in females (Additional file 6: Figure S2B, Additional file 4: Table S3). NIS immunostaining, in contrast, increased by gestation age in males and remained constant in females (Additional file 6: Figure S2B, Additional file 4: Table S3). Calcitonin immunostaining scores on fetal thyroid tissues increased with fetal age and the FOXA2 immunostaining score remained constant in males and females (Additional file 4: Table S3).

Levels of transcripts encoding *AHR*, *BAX*, *BCL*, *ESR1*, *FOXA2*, and *TPO* increased with fetal age, irrespective of fetal sex, smoke exposure, or maternal BMI (Additional file 5: Table S4 and Additional file 7: Table S5). In contrast, levels of *GATA4* transcripts decreased with fetal age. Male fetal thyroids had lower levels of *AHR*, *ARNT*, and *AR* transcripts than females while the *BAX*/*BCL2* ratio increased with fetal age in males (Additional file 6: Figure S2C, Additional file 5: Table S4 and Additional file 7: Table S5), indicative of increased apoptosis.

### Maternal smoke exposure involves sex-specific changes in fetal thyroid-related endocrine functions and development

**Fig. 2:**
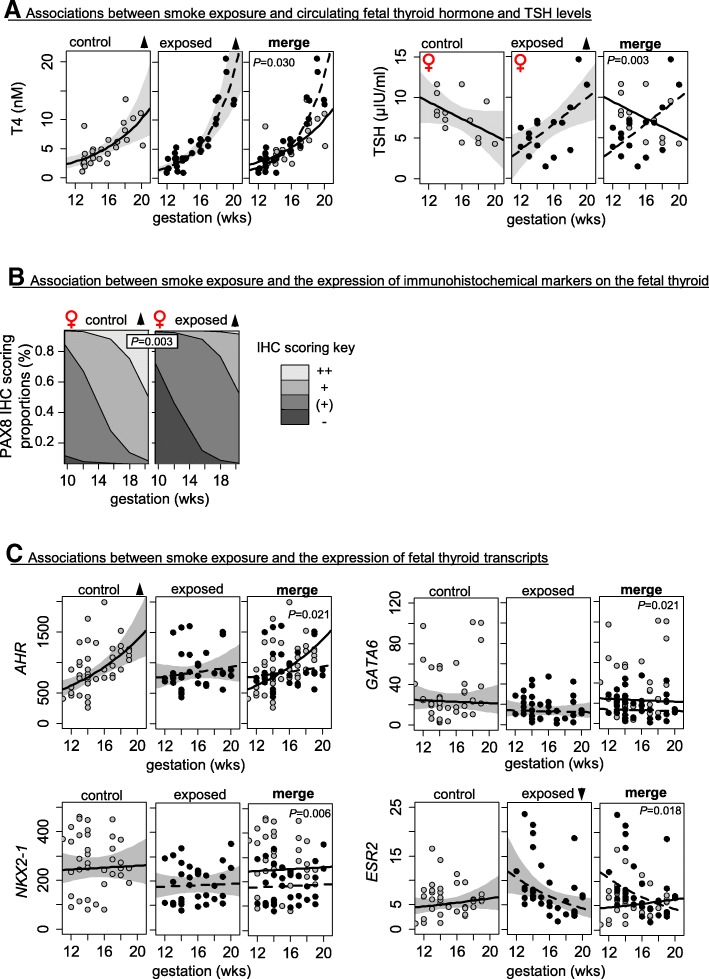
Statistically significant (*P* < 0.05) effects of maternal smoke exposure on **a** circulating hormones, **b** immunohistochemistry scoring proportions, and **c** key thyroid transcripts. Shaded areas in **a** and **c** represent 95% confidence intervals. Immunohistochemistry scores in **b** are shown as stacked percentages for each scoring (“−, unstained” < “(+)” < “+” < “++, more stained”) across gestation. Arrowheads indicate significant (*P* < 0.05) increase (▲) or decrease (▼) by gestational age. *P* values associated with changes in relation to smoke exposure are provided in the merged graph panels

Maternal smoke exposure accelerated the developmental increase of T4 regardless of fetal sex, and this was associated with a developmental increase in TSH levels in smoke-exposed female fetuses (Fig. 2a, Table 1). TSH levels did not, however, correlate with fetal plasma cotinine levels in either sex (Additional file 8: Figure S3A*i*) and did not correlate with T4 in males (Additional file 8: Figure S3A*ii*). In contrast, fetal TSH levels did show a negative correlation with fetal T4 levels in control females and a positive correlation in smoke-exposed females (Additional file 8: Figure S3A*ii*). This developmentally dependent increase in TSH in smoke-exposed females was evident in both the BMI < 25 and BMI > 25 subgroups (Additional file 8: Figure S3C*ii*). Maternal smoke exposure did not affect thyroid weight and thyroid morphology in either sex (Additional file 4: Table S3), but reduced PAX8 immunostaining scores across gestation in smoke-exposed females (Fig. 2b, Additional file 4: Table S3). Smoke exposure also abolished the normal developmental increase in *AHR* transcripts, caused an age-dependent reduction in *ESR2* transcripts, and reduced *NKX2-1*, *GATA6*, and *TSHR* transcript levels in thyroid tissue of both sexes (Fig. 2c, Additional file 5: Table S4).

### High maternal BMI associates with sex-specific alterations in fetal thyroid endocrinology, morphology, and expression of developmental markers

**Fig. 3:**
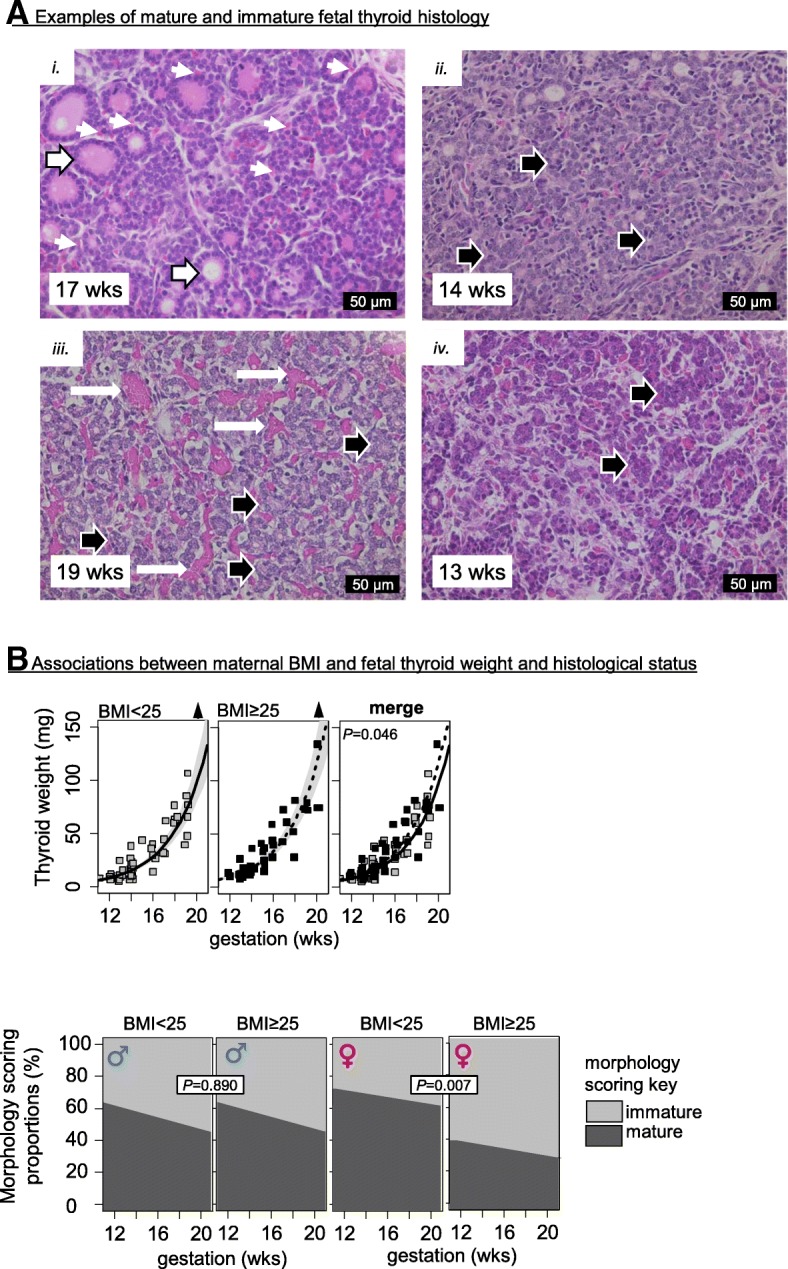
**a** Representative examples of mature and immature thyroid histology: (*i*) image of a normal thyroid morphology showing well-developed angiofollicular units with colloid-filled thyroid follicles (large arrows) and surrounding capillaries (small arrows); (*ii–iv*) examples for thyroid immaturity with lack of angiofollicular units as shown by unorganized arrangement of thyroid cells without follicle formation (black arrows in *ii*, *iii*, *iv*), the presence of numerous dilated blood vessels (white arrows in *iii*), and thyroid cell nests without apicobasal orientation embedded in increased stroma (large arrows in *iv*). Gestational ages (weeks) and scale bars (μm) are shown on the bottom left and right side of each image respectively. **b** Statistically significant (*P* < 0.05) changes in thyroid weight and histological morphology scoring proportions (immature vs mature) in relation to maternal BMI. Shaded areas in the thyroid weight plots represent 95% confidence intervals. Arrowheads indicate significant (*P* < 0.05) increase (▲) or decrease (▼) by gestational age

**Fig. 4:**
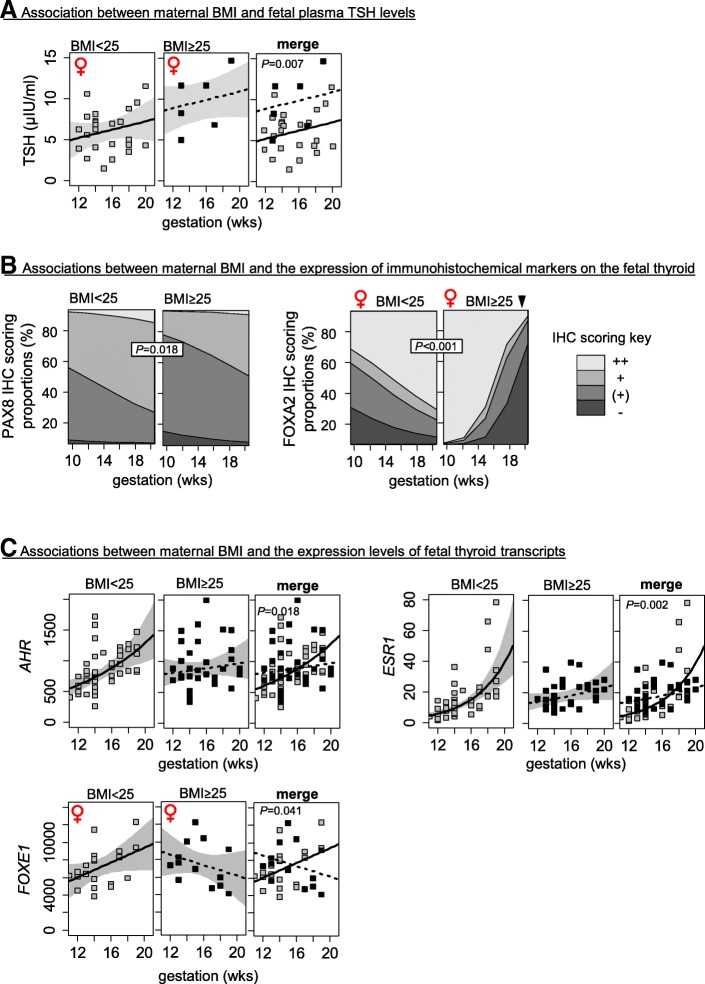
Statistically significant (*P* < 0.05) effects of maternal BMI on **a** circulating hormones, **b** immunohistochemistry scores, and **c** transcripts. Shaded areas in **a** and **c** represent 95% confidence intervals. Immunohistochemistry scoring proportions in **b** are shown as stacked percentages for each scoring (“−, unstained” < “(+)” < “+” < “++, more stained”) across gestation. Arrowheads indicate significant (*P* < 0.05) increase (▲) or decrease (▼) by gestational age. *P* values associated with changes in relation to smoke exposure are provided in the merged graph panels

High maternal BMI was associated with increased thyroid weight and with sex-specific alterations in thyroid morphology, with female thyroids from mothers with BMI ≥ 25 exhibiting a higher percentage of immature morphology (Fig. 3b, Additional file 4: Table S3). The fetal thyroid tissues that were scored as immature showed alterations resembling delayed development of angio-follicular units, with areas of dense thyroid tissue composed of predominantly immature thyroid follicles, scarcity of organized thyroid follicles, and marked blood vessel dilations (Fig. 3a). Maternal BMI status did not affect T3 and T4 levels but increased TSH levels in female fetuses (Fig. 4a, Table 1) with maternal BMI positively correlated with fetal TSH levels in females (*P* = 0.0004, *R*^*2*^ = 0.37, Additional file 8: Figure S3B). To further understand the relationship between TSH, BMI, and smoke exposure in the females, the TSH levels were examined in relation to BMI in the control and smoke-exposed populations separately (Additional file 8: Figure S3C). The results showed that, irrespective of smoke exposure, increased maternal BMI was associated with increased fetal TSH (controls: *P* = 0.049, *R*^*2*^ = 0.28; smoke-exposed: *P* = 0.003, *R*^*2*^ = 0.48) and that, irrespective of maternal weight, smoke exposure was associated with increased developmental trajectory of TSH (Additional file 8: Figure S3C*iii-iv*).

Fetal thyroid tissues from mothers of BMI ≥ 25 had reduced PAX8 scores while female thyroid tissues from mothers of BMI ≥ 25 were more likely to have lower FOXA2 immunohistochemical scores (Fig. 4b, Additional file 4: Table S3). Maternal BMI ≥ 25 was associated with altered development of *AHR* and *ESR1* transcript levels in both sexes (Fig. 4c, Additional file 7: Table S5) and reversed the developmental trajectory of *FOXE1* transcripts in female fetuses (Fig. 4c, Additional file 7: Table S5).

## Discussion

The developmental processes involved in normal fetal thyroid function are complex and essential for fetal brain development and in utero growth [1]. The second trimester marks a critical developmental window when the fetal thyroid gland begins to assume its critical endocrine functions. Two major maternal lifestyle factors, smoking and being overweight, have been implicated in the disruption of thyroid function in the neonate [18–20]. However, the mechanisms linking maternal smoking and increased maternal weight to thyroid disruption are largely unknown. It is equally unclear whether these maternal risk factors cause persistent changes in human fetal thyroid function that will contribute to the development of diseases, such as metabolic syndrome, in adulthood [6, 7]. Here, we have performed an in-depth examination of thyroid morphology, fetal thyroid endocrine function, and associated protein and transcript expression during development in the second trimester under normal conditions and with altered maternal lifestyle. We report that both maternal smoke exposure and overweight/obese maternal status during the critical second trimester are associated with significant alterations in morphology and endocrine functions of the fetal thyroid in a sex-specific manner.

### The roles of age and sex in human fetal thyroid development

The lack of developmental changes in *SLC5A5*, *TSHR*, *PAX8*, *NKX2-1*, *FOXE* transcript levels; the developmental increase of *TPO* transcripts; the immunohistochemical localization of TTF1; and the developmental shift of NIS staining from perinuclear to basolateral observed here (Additional file 5: Table S4 and Additional file 7: Table S5; Fig. 1) are consistent with previous observations on the human fetal thyroid [28]. In addition, data described here show that the human fetal thyroid gland is sexually dimorphic and that the sexes respond differently to maternal smoking or increased BMI. The developmentally regulated transcription factors *FOXA1*, *FOXA2*, *GATA4*, *GATA6*, *PAX8*, *NKX2-1*, and *SOX17* did not exhibit any statistically significant sex-specific differences in transcription (Additional file 4: Table S4 and Additional file 7: Table S5). Nevertheless, the male thyroids tended to show a more immature morphology (did not reach statistical significance), irrespective of smoke exposure or maternal BMI (Fig. 3b, Additional file 4: Table S3). This trend to an underlying phenotypic difference between the sexes requires further investigation. However, we observed two potential contributing factors: (i) immunoreactivity levels of PAX8, which is essential for the formation and organization of follicular cells in the thyroid gland [29, 30], were constant between 11 and 21 weeks in males but showed an age-dependent increase in female thyroid glands (Additional file 6: Figure S2B); (ii) the transcriptional expression of both *AHR* and *ARNT*, two key mediators of xenobiotic and detoxification responses, were reduced in male thyroid tissues (Additional file 5: Table S4 and Additional file 7: Table S5), suggesting that the male thyroids are more susceptible to potentially harmful environmental contaminants. Male fetuses also had higher circulating T3 levels and a higher T3/T4 ratio which may be the consequence of both increased NIS levels during gestation and increased rates of T4-to-T3 deiodination in fetal tissues (Additional file 6: Figure S2A and S2B). Consistent with the latter, male fetuses are known to have a higher metabolic rate than females [31]. Overall, the data suggest more rapid organizational development in the female with greater sensitivity to disruption and changes in thyroid hormones in the males.

### The effect of maternal smoking in the fetal thyroid gland

Of the key thyroid transcripts levels quantified, maternal smoking downregulated the transcription factors *GATA6* and *NKX2-1* in both male and female thyroids (Fig. 2c, Additional file 5: Table S4), suggesting a disruption of fetal thyroid development, which may affect T4 production and release. TTF1 (gene name: *NKX2-1*) is under the control of GATA6 [32], and a GATA6-TTF1 protein complex directly affects the expression of various genes [33]. The TTF1 transcription factor also controls the expression of *TSHR* [34], and here, the reduced thyroid *TSHR* transcript (Additional file 5: Table S4) levels is a possible consequence of reduced *GATA6* and *NKX2-1* transcripts. Even though the downregulation of *GATA6* and/or *NKX2-1* transcripts by maternal smoking during the second trimester may contribute to neonatal phenotypes such as thyroid enlargement and/or reduction of TSH levels [18, 19], it is uncertain whether the small changes for both *GATA6* and *NKX2-1* (Additional file 5: Table S4) can, alone, account for profound alterations on the thyroid.

Since the limited volumes of fetal plasma available for the analyses performed in this study precluded measurement of free T3 (fT3) and free T4 (fT4) in the fetal circulation, we also measured fetal circulating binding proteins: ALB, TTR, and TBG (Table 1). Levels of ALB, TTR, and TBG in the fetus were unaffected by maternal smoking (Table 1), and so it is likely that changes in total T3 and total T4 levels measured here reflect changes in fT3 and fT4. As T3 and the T3/T4 ratio were unaffected by smoke exposure, the increase in the developmental trajectory of T4 is unlikely, however, to reflect altered deiodination to T3 (Fig. 2a, Table 1). Instead, it may suggest an increased sensitivity of the fetal thyrocytes to TSH, although *THSR* transcript levels are reduced in smoke-exposed fetuses (Additional file 5: Table S4). It is also possible that the apparent age-dependent increase in TSH levels in smoke-exposed female fetuses (Fig. 2a) may be because TSH is initially suppressed by maternal smoking (Fig. 2a, Table 1). The age-dependent increase in TSH levels in smoke-exposed female fetuses (Fig. 2a) may account for the accelerated rate of T4 production, although no such effect was observed in males (Table 1), suggesting a complex regulatory network. It is likely, therefore, that the hypothalamic-pituitary-thyroid axis (HPT) in the fetus is not only sexually dimorphic but also affected by smoke exposure in a sex-specific manner. The sexual dimorphism of the HPT axis and in particular TSH levels are influenced by many factors, including sex steroids and adrenal function, and are subject to diurnal variation, as has previously been demonstrated in rats and humans [35–39]. Our studies show that *AR* is expressed in the human fetal thyroid and that male fetal thyroids have reduced *AR* transcript levels (Additional file 6: Figure S2C, Additional file 5: Table S4 and Additional file 7: Table S5). In addition, smoke exposure altered the developmental trajectory of thyroid *ESR2* transcripts (Fig. 2c, Additional file 5: Table S4), suggesting that sex-specific effects of maternal smoking on the fetal thyroid may be mediated by fetal sex hormones. In support of this, non-smoke-exposed female fetuses show an inverse relationship between TSH and T4 in agreement with current population data [40], which is absent in males and is reversed in smoke-exposed females (Additional file 8: Figure S3A*ii*). Finally, our studies show that maternal smoking reduced immunoreactive PAX8 in female fetuses only, which may affect the differentiation as well as the TSH responsiveness of hormone-producing fetal thyrocytes.

### The sex-specific effects of maternal overweight/obesity status on the fetal thyroid

In this study, a maternal BMI above 25 was associated with a small but significant increase in fetal thyroid weights of both sexes although total circulating thyroid hormone levels remained unchanged (Fig. 2b, Table 1, Additional file 4: Table S3). High maternal BMI coincided with an immature histological phenotype, preferentially affecting the thyroid gland of female fetuses (Fig. 2b). Dysregulation of morphogenic transcription factors such as FOXA1, FOXA2, and FOXE1 can cause morphological deviations, and in this study, the immunoreactivity of FOXA2 and the expression of *FOXE1* were affected in female thyroids from mothers with BMI ≥ 25 (Fig. 4b, c, Additional file 4: Table S3 and Additional file 7: Table S5). It is possible that the developmental changes in FOXA2 and *FOXE1* expression control thyroid folliculogenesis and that deviations seen in the female fetuses from mothers of BMI ≥ 25 contributed to the perturbed thyroid folliculogenesis. The FOXA2 transcription factor is expressed in the C cell precursors of the mouse fetal thyroid, and it may modulate the expression of TTF1 while also playing a role in thyroid regeneration [41–43]. FOXA2 distribution in the human thyroid suggests similar developmental functions, with FOXA2 staining sometimes resembling the distribution of calcitonin-positive C-cells (Fig. 1e, middle panel compared to Fig. 1c, right panel).

As in the case of smoke exposure, changes in the expression of fetal thyroid *ESR1* transcripts in overweight mothers (Fig. 4c, Additional file 7: Table S5) may have affected responsiveness to sex steroids and may, therefore, contribute to sex-specific responses to maternal BMI. The increased circulating TSH levels, with unaffected thyroid hormone levels, in female fetuses from mothers with BMI ≥ 25 are consistent with data that have been reported for overweight and obese adults [44, 45]. Taken together, this suggests altered hypothalamic-pituitary function and/or reduced fetal thyroid sensitivity to TSH in female fetuses.

### Overall conclusions

The fetal thyroid gland plays an essential role in the control of fetal metabolic rate, cardiac output, and brain development while normal thyroid gland function is essential for post-natal health. Maternal lifestyle factors, such as smoking and increased BMI, are associated with adverse outcomes for the child, and here, we examined the effects of those factors on the human fetal thyroid system. Unfortunately, this study lacks knowledge of the thyroid status of the consenting women in this study which could have explained, at least in part, fetal thyroid alterations observed here in response to smoking and BMI. Nevertheless, this is the first study to demonstrate adverse effects on the developing thyroid system associated with maternal lifestyle during the second trimester. Critically, this is the period when the fetal thyroid begins to secrete hormones, and it is likely that changes in thyroid function at this time will have a significant impact on fetal development with implications for the fetal programming of adult diseases.

## Additional files

Additional file 1: **Figure S1.** Boxplots across the fetal age in relation (*i*) to maternal smoke exposure (SE) or (*ii*) maternal BMI status in either sex and (*iii*) of BMI values across maternal smoke exposure in either sex for the samples used for **A.** combined right and left gland weight; **B.** hormonal measurements; **C.** circulating thyroid hormone-binding proteins; **D.** H&E thyroid morphology scoring; **E.** Thyroid immunohistochemical scoring; and **F.** transcript measurements. Asterisks (*) indicate significant mean differences (*P* < 0.05) between the groups compared (bracketed) C: non-smoke exposed control; SE: smoke-exposed. (PDF 434 kb)

Additional file 2: **Table S1.** Antibodies, dilution used, and company information. (DOCX 15 kb)

Additional file 3: **Table S2.** Gene names and primer sequences used for real-time quantitative PCR. Housekeeping genes are shown in bold. (DOCX 20 kb)

Additional file 4: **Table S3.** Significance (*P* values) of associations between fetal age, sex, smoke exposure, high maternal BMI, and their interactions (2-way and 3-way analyses), and thyroid weight, morphology and immunostaining. Statistically significant differences (DOCX 29 kb)

Additional file 5: **Table S4.** Significance (*P* values) of associations between fetal age, sex, smoke exposure, and their interactions (2-way and 3-way analyses), and normalized fetal thyroidal transcripts. Data were log-transformed for those cases where model residuals departed from normality. Statistically significant differences (*P* < 0.05) are shown in bold. C: controls; SE: smoke-exposed. N/A, not applicable. (DOCX 30 kb)

Additional file 6: **Figure S2.** Statistically significant (*P* < 0.05) effects of fetal sex on **A.** circulating hormones, **B.** immunohistochemistry scores, and **C.** transcripts. Shaded areas in **A** and **C** represent 95% confidence intervals. Immunohistochemistry scoring proportions in **B** are shown as stacked percentages for each scoring (“−, unstained” < “(+)” < “+” < “++, more stained”) across gestation. Arrowheads indicate significant (*P* < 0.05) increase (▲) or decrease (▼) by gestational age. *P* values associated with changes in relation to smoke exposure are provided in the merged graph panels. (PDF 474 kb)

Additional file 7: **Table S5.** Significance (*P* values) of associations between fetal age, sex, maternal BMI, and their interactions (2-way and 3-way analyses), and normalized human fetal thyroid transcripts. Data were log-transformed for those cases where model residuals departed from normality. Statistically significant differences (*P* < 0.05) are shown in bold. N/A: not applicable. (DOCX 32 kb)

Additional file 8: **Figure S3.** **A.** Correlations among TSH, cotinine, and T4 in the human fetal circulation. **B.** Correlation between fetal TSH and maternal BMI (continuous). **C.** Associations between smoking, maternal BMI, and fetal TSH levels in the female fetuses. In **C***i-ii*, the females were stratified according to smoke exposure and the effect of age and BMI (categorical) on TSH was examined. In **C***iii-iv*, TSH levels were correlated to BMI (continuous) in the control and smoke-exposed females. Pearson’s correlation *P* values in **A**, **B**, **C***iii*, and **C***iv* are given within the graphs, including *R*^*2*^ values in the cases of statistically significance (*P* < 0.05). Arrowheads above the graphs indicate significant (*P* < 0.05) increase (▲) or decrease (▼). (PDF 451 kb)

## Abbreviations

ACTH: Adrenocorticotropic hormone
*AHR*: Aryl hydrocarbon receptor
ALB: Albumin
*ARNT*: Aryl hydrocarbon receptor nuclear translocator
*B2M*: Beta-2 microglobulin
*BAX*: BCL2-associated X
*BCL2*: B-cell CLL/lymphoma 2
BMI: Body mass index
CNTF: Ciliary neurotrophic factor
DAB: 3,3′-diaminobenzidine tetrahydrochloride
*ESR1*: Estrogen receptor 1
*ESR2*: Estrogen receptor 2
FOXA1: Forkhead box A1
FOXA2: Forkhead box A2
FOXE1: Forkhead box E1
fT3: Free triiodothyronine
fT4: Free thyroxine
*GATA4*: GATA binding protein 4
*GATA6*: GATA binding protein 6
GH: Growth hormone
GRP: Gastrin-releasing peptide
HKG: House-keeping gene
HPT: Hypothalamic pituitary thyroid
NHS: National Health Service
NIS: Sodium-iodide symporter
*NKX2-1*: NK2 homeobox 1
PAX8: Paired box 8
*PMM1*: Phosphomannomutase 1
*SDHA*: Succinate dehydrogenase complex flavoprotein subunit A
*SFRS4*: Serine and arginine-rich splicing factor 4
SOX17: SRY-box 17
T3: Triiodothyronine
T4: Thyroxine
TBG: Thyroxine-binding globulin
*TBP*: TATA-box binding protein
TBST: Tris-based saline with 0.05% Tween-20
TSH: Thyroid-stimulating hormone
TTF1: Thyroid transcription factor 1
TTR: Transthyretin

## References

[1] Bernal J (2005). Thyroid hormones and brain development. Vitam Horm.

[2] Morreale de Escobar G, Obregón MJ, Escobar del Rey F (1987). Fetal and maternal thyroid hormones. Horm Res.

[3] Vulsma T, Gons MH, de Vijlder J (1989). Maternal-fetal transfer of thyroxine in congenital hypothyroidism due to a total organification defect or thyroid dysgenesis. N Engl J Med.

[4] Barker D (1999). Early growth and cardiovascular disease. Arch Dis Child.

[5] Tappin DM, MacAskill S, Bauld L, Eadie D, Shipton D, Galbraith L (2010). Smoking prevalence and smoking cessation services for pregnant women in Scotland. Subst Abuse Treat Prev Policy.

[6] Behl M, Rao D, Aagaard K, Davidson TL, Levin ED, Slotkin TA (2013). Evaluation of the association between maternal smoking, childhood obesity, and metabolic disorders: a national toxicology program workshop review. Environ Health Perspect.

[7] Högberg L, Cnattingius S, Lundholm C, D'Onofrio BM, Långström N, Iliadou AN (2012). Effects of maternal smoking during pregnancy on offspring blood pressure in late adolescence. J Hypertens.

[8] Mamsen LS, Lutterodt MC, Andersen EW, Skouby SO, Sørensen KP, Andersen CY (2010). Cigarette smoking during early pregnancy reduces the number of embryonic germ and somatic cells. Hum Reprod.

[9] Jensen TK, Jørgensen N, Punab M, Haugen TB, Suominen J, Zilaitiene B (2004). Association of in utero exposure to maternal smoking with reduced semen quality and testis size in adulthood: a cross-sectional study of 1,770 young men from the general population in five European countries. Am J Epidemiol.

[10] Tweed S, Bhattacharya S, Fowler PA (2017). Effects of maternal smoking on offspring reproductive outcomes: an intergenerational study in the North East of Scotland. Human Reproduction Open.

[11] Weissman MM, Warner V, Wickramaratne PJ, Kandel DB (1999). Maternal smoking during pregnancy and psychopathology in offspring followed to adulthood. J Am Acad Child Adolesc Psychiatry.

[12] Flegal KM, Carroll MD, Kit BK, Ogden CL (2012). Prevalence of obesity and trends in the distribution of body mass index among US adults, 1999-2010. JAMA.

[13] Heslehurst N, Rankin J, Wilkinson JR, Summerbell CD (2010). A nationally representative study of maternal obesity in England, UK: trends in incidence and demographic inequalities in 619 323 births, 1989-2007. Int J Obes.

[14] Reynolds RM, Allan KM, Raja EA, Bhattacharya S, McNeill G, Hannaford PC (2013). Maternal obesity during pregnancy and premature mortality from cardiovascular event in adult offspring: follow-up of 1 323 275 person years. BMJ.

[15] Fraser A, Tilling K, Macdonald-Wallis C, Sattar N, Brion MJ, Benfield L (2010). Association of maternal weight gain in pregnancy with offspring obesity and metabolic and vascular traits in childhood. Circulation.

[16] Ekström S, Magnusson J, Kull I, Lind T, Almqvist C, Melén E (2015). Maternal body mass index in early pregnancy and offspring asthma, rhinitis and eczema up to 16 years of age. Clin Exp Allergy.

[17] Wang Y, Tang S, Xu S, Weng S, Liu Z (2016). Maternal body mass index and risk of autism spectrum disorders in offspring: a meta-analysis. Sci Rep.

[18] Chanoine JP, Toppet V, Bourdoux P, Spehl M, Delange F (1991). Smoking during pregnancy: a significant cause of neonatal thyroid enlargement. Br J Obstet Gynaecol.

[19] Shields B, Hill A, Bilous M, Knight B, Hattersley AT, Bilous RW, Vaidya B (2008). Cigarette smoking during pregnancy is associated with alterations in maternal and fetal thyroid function. J Clin Endocrinol Metab.

[20] Kahr MK, Antony KM, DelBeccaro M, Hu M, Aagaard KM, Suter MA (2016). Increasing maternal obesity is associated with alterations in both maternal and neonatal thyroid hormone levels. Clin Endocrinol.

[21] Fowler PA, Cassie S, Rhind SM, Brewer MJ, Collinson JM, Lea RG, Baker PJ, Bhattacharya S, O'Shaughnessy PJ (2008). Maternal smoking during pregnancy specifically reduces human fetal desert hedgehog gene expression during testis development. J Clin Endocrinol Metab.

[22] Audet-Delage Y, Ouellet N, Dallaire R, Dewailly E, Ayotte P (2013). Persistent organic pollutants and transthyretin-bound thyroxin in plasma of Inuit women of childbearing age. Environ Sci Technol.

[23] Cox J, Mann M (2008). MaxQuant enables high peptide identification rates, individualized p.p.b.-range mass accuracies and proteome-wide protein quantification. Nat Biotechnol.

[24] Cox J, Hein MY, Luber CA, Paron I, Nagaraj N, Mann M (2014). Accurate proteome-wide label-free quantification by delayed normalization and maximal peptide ratio extraction, termed MaxLFQ. Mol Cell Proteomics.

[25] O'Shaughnessy PJ, Monteiro A, Bhattacharya S, Fowler PA (2011). Maternal smoking and fetal sex significantly affect metabolic enzyme expression in the human fetal liver. J Clin Endocrinol Metab.

[26] Vandesompele J, De Preter K, Pattyn F, Poppe B, Van Roy N, De Paepe A, Speleman F (2002). Accurate normalization of real-time quantitative RT-PCR data by geometric averaging of multiple internal control genes. Genome Biol.

[27] Andersen CL, Jensen JL, Ørntoft TF (2004). Normalization of real-time quantitative reverse transcription-PCR data: a model-based variance estimation approach to identify genes suited for normalization, applied to bladder and colon cancer data sets. Cancer Res.

[28] Szinnai G, Lacroix L, Carré A, Guimiot F, Talbot M, Martinovic J (2007). Sodium/iodide symporter (NIS) gene expression is the limiting step for the onset of thyroid function in the human fetus. J Clin Endocrinol Metab.

[29] Mansouri A, Chowdhury K, Gruss P (1998). Follicular cells of the thyroid gland require Pax8 gene function. Nat Genet.

[30] Koumarianou P, Goméz-López G, Santisteban P (2017). Pax8 controls thyroid follicular polarity through cadherin-16. J Cell Sci.

[31] Lampl M, Gotsch F, Kusanovic JP, Gomez R, Nien JK, Frongillo EA, Romero R (2010). Sex differences in fetal growth responses to maternal height and weight. Am J Hum Biol.

[32] Shaw-White JR, Bruno MD, Whitsett JA (1999). GATA-6 activates transcription of thyroid transcription factor-1. J Biol Chem.

[33] Liu C, Glasser SW, Wan H, Whitsett JA (2002). GATA-6 and thyroid transcription factor-1 directly interact and regulate surfactant protein-C gene expression. J Biol Chem.

[34] Moeller LC, Kimura S, Kusakabe T, Liao XH, Van Sande J, Refetoff S (2003). Hypothyroidism in thyroid transcription factor 1 haploinsufficiency is caused by reduced expression of the thyroid-stimulating hormone receptor. Mol Endocrinol.

[35] Donda A, Reymond F, Rey F, Lemarchand-Béraud T (1990). Sex steroids modulate the pituitary parameters involved in the regulation of TSH secretion in the rat. Acta Endocrinol.

[36] Christianson D, Roti E, Vagenakis AG, Braverman LE (1981). The sex-related difference in serum thyrotropin concentration is androgen mediated. Endocrinology.

[37] Ling C, Sun Q, Khang J, Felipa Lastarria M, Strong J, Stolze B (2018). Does TSH reliably detect hypothyroid patients?. Ann Thyroid Res.

[38] Hannah-Shmouni F, Soldin SJ (2017). Thyroid hormone therapy for older adults with subclinical hypothyroidism. N Engl J Med.

[39] Sheikh SI, Parikh TP, Kushchayeva Y, Stolze B, Masika LS, Ozarda Y (2018). TSH should not be used as a single marker of thyroid function. Annals Thyroid Res.

[40] Hadlow NC, Rothacker KM, Wardrop R, Brown S, Lim EM, Walsh JP (2013). The relationship between TSH and free T4 in a large population is complex and nonlinear and differs by age and sex. J Clin Endocrinol Metab.

[41] Johansson E, Andersson L, Örnros J, Carlsson T, Ingeson-Carlsson C, Liang S (2015). Revising the embryonic origin of thyroid C cells in mice and humans. Development.

[42] Dame K, Cincotta S, Lang AH, Sanghrajka RM, Zhang L, Choi J (2017). Thyroid progenitors are robustly derived from embryonic stem cells through transient, developmental stage-specific overexpression of Nkx2-1. Stem Cell Reports.

[43] Ozaki T, Matsubara T, Seo D, Okamoto M, Nagashima K, Sasaki Y (2012). Thyroid regeneration: characterization of clear cells after partial thyroidectomy. Endocrinology.

[44] Muscogiuri G, Sorice GP, Mezza T, Prioletta A, Lassandro AP, Pirronti T (2013). High-normal TSH values in obesity: is it insulin resistance or adipose tissue’s guilt?. Obesity (Silver Spring).

[45] Ruhla S, Weickert MO, Arafat AM, Osterhoff M, Isken F, Spranger J (2010). A high normal TSH is associated with the metabolic syndrome. Clin Endocrinol.

