# Supplementary material for: Maternal smoking and high BMI disrupt thyroid gland development
Source: BMC Med. 2018 Oct 23;16:194. doi: 10.1186/s12916-018-1183-7 (PMC6198368; doi:10.1186/s12916-018-1183-7)

Supplementary Figure 1

A. Spread of samples used for fetal thyroid weight measurements

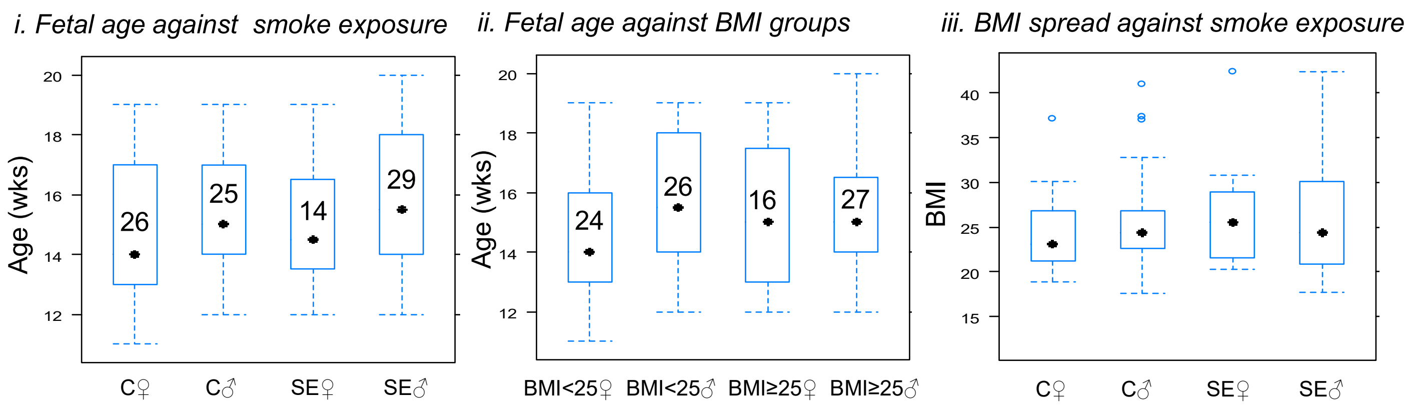

B. Spread of samples used for fetal plasma hormonal measurements

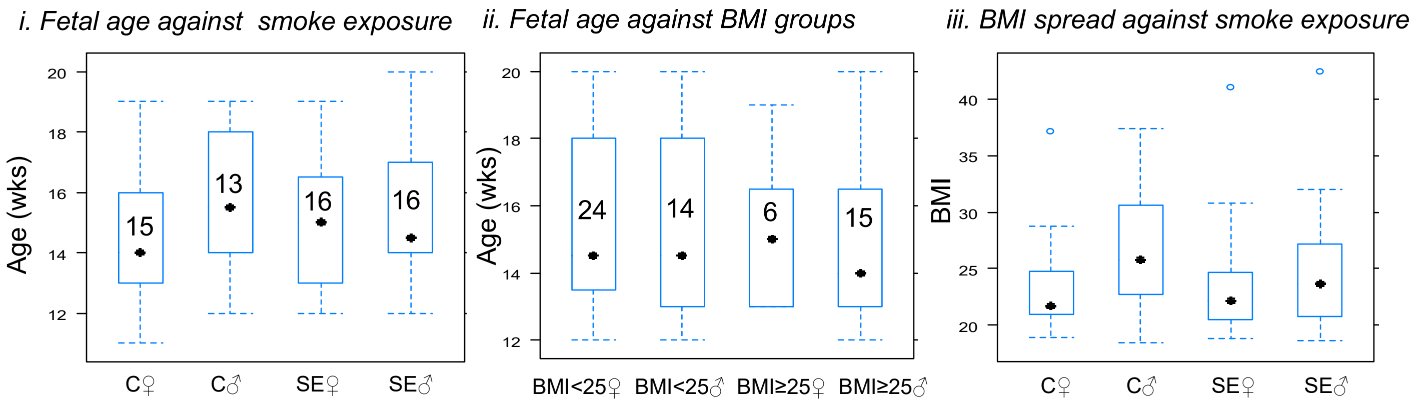

C. Spread of samples used for determination of circulating thyroid hormone-binding proteins

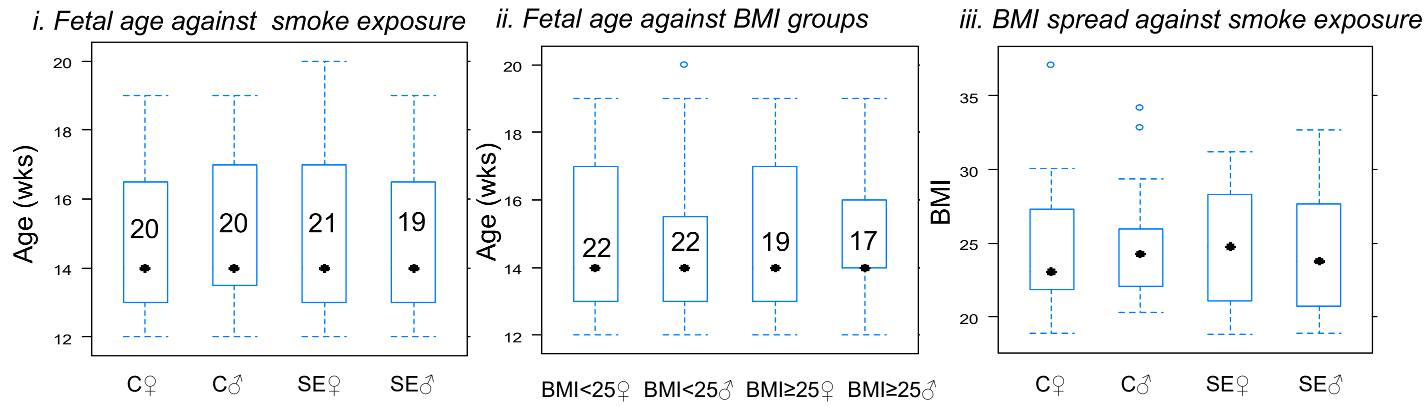

D. Spread of samples used for fetal thyroid H&E morphology scoring

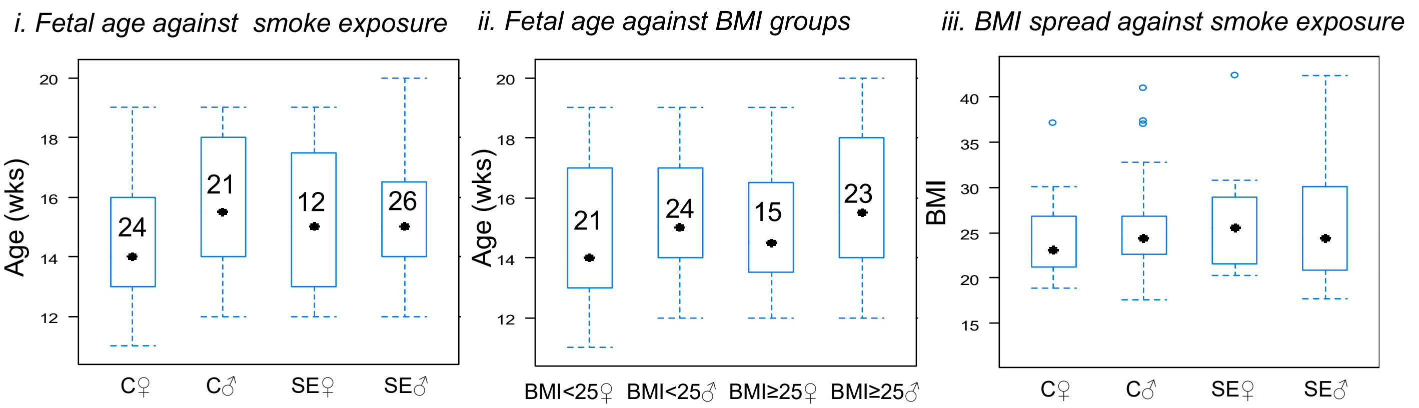

Supplementary Figure 1 continues on the next page

Supplementary Figure 1 (continued)

E. Spread of samples used for fetal thyroid immunohistochemical scoring

i. Fetal age against smoke exposure

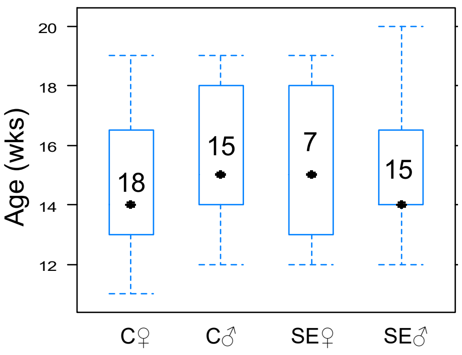

ii. Fetal age against BMI groups

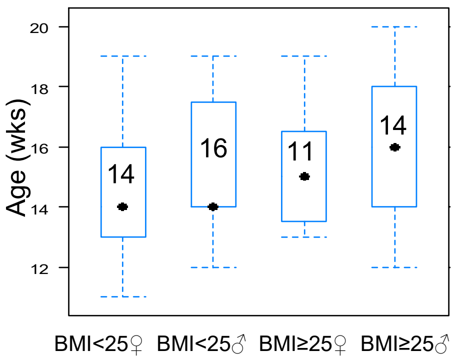

iii. BMI spread against smoke exposure

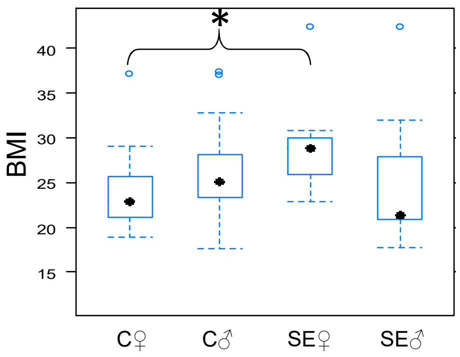

F Spread of samples used for fetal thyroid transcript measurements

i. Fetal age against smoke exposure

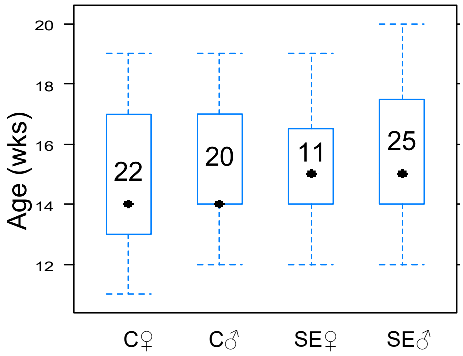

ii. Fetal age against BMI groups

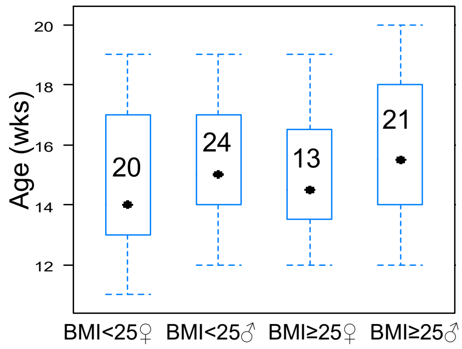

iii. BMI spread against smoke exposure

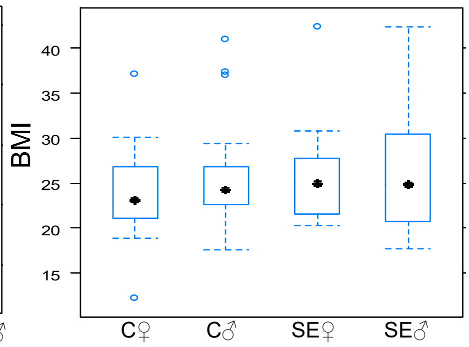

Supplement: Supplementary file 1 — Figure S1. Boxplots across the fetal age in relation (i) to maternal smoke exposure (SE) or (ii) maternal BMI status in either sex and (iii) of BMI values across maternal smoke exposure in either sex for the samples used for A. combined right and left gland weight; B. hormonal measurements; C. circulating thyroid hormone-binding proteins; D. H&E thyroid morphology scoring; E. Thyroid immunohistochemical scoring; and F. transcript measurements. Asterisks (*) indicate significant mean differences (P < 0.05) between the groups compared (bracketed) C: non-smoke exposed control; SE: smoke-exposed. (PDF 434 kb) [file 12916_2018_1183_MOESM1_ESM.pdf]
