# Supplementary material for: Maternal smoking and high BMI disrupt thyroid gland development
Source: BMC Med. 2018 Oct 23;16:194. doi: 10.1186/s12916-018-1183-7 (PMC6198368; doi:10.1186/s12916-018-1183-7)
Supplement: Supplementary file 2 — Table S1. Antibodies, dilution used, and company information. (DOCX 15 kb) [file 12916_2018_1183_MOESM2_ESM.docx]

**Additional file 2: Table S1:** Antibodies, dilution used and company information.

| **Antibody** | **Dilution** | **Species raised** | **Catalogue** | **Company** |
| --- | --- | --- | --- | --- |
| anti-PAX8 | 1:100 | Mouse | ACI 438 | Biocare Medical, Pacheco, CA, USA |
| anti-TTF1 | 1:250 | Rabbit monoclonal | LS-C154673 | LifeSpan Biosciences, Seattle, WA, USA |
| anti-NIS | 1:200 | Mouse | MA5-12308, clone FP5A | Thermo Fisher, Rockford, IL, USA |
| anti-FOXA2 | 1:250 | Mouse | H00003170-M12 | Novus Biologicals, Oakville, ON, Canada |
| anti-Calcitonin | 1:200 | Rabbit Polyclonal | PA5-16464 | Thermo Fisher Scientific, Rockford, IL, USA |
| HRP-anti-mouse | 1:200 | Goat | 115-035-003 | Jackson Immunoresearch, West Grove, PA, USA |
| HRP-anti-mouse | 1:200 | Goat | 111-035-144 | Jackson Immunoresearch, West Grove, PA, USA |
