# Supplementary material for: Maternal smoking and high BMI disrupt thyroid gland development
Source: BMC Med. 2018 Oct 23;16:194. doi: 10.1186/s12916-018-1183-7 (PMC6198368; doi:10.1186/s12916-018-1183-7)
Supplement: Supplementary file 3 — Table S2. Gene names and primer sequences used for real-time quantitative PCR. Housekeeping genes are shown in bold. (DOCX 20 kb) [file 12916_2018_1183_MOESM3_ESM.docx]

| **Gene** | **Gene name** | **Genbank** | **Forward / reverse primer** |
| --- | --- | --- | --- |
| *AHR* | Aryl hydrocarbon receptor | NM_001621 | AATACAGAGTTGGACCGTTTGGCTAGC / TGGCCTCCGTTTCTTTCAGTAGGG |
| *AR* | Androgen receptor | NM_001011645 | CTTCTGGGTGTCACTATGGAGCTCTCA  / AACATTTCCGAAGACGACAAGATGGAC |
| *ARNT* | Aryl hydrocarbon receptor nuclear translocator | NM_001668 | CCTCTGGAAACTCTGGACCTGGAATTC / TGGCAAACCGCTCCTTATCGTTAG/ |
| *BAX* | BCL2-associated X protein | NM_004324 | TGGACCCGGTGCCTCAGGAT / AAACATGTCAGCTGCCACTCGGA |
| *BCL2* | B-cell CLL/lymphoma 2 | NM _000633 | AAAAGGAAACTTGACAGAGGATCATGCTG / TCCCCCTTGGCATGAGATGC |
| *ESR1* | Estrogen receptor 1 | NM_000125 | ATTGGTCTCGTCTGGCGCTCC / CCCTGCAGATTCATCATGCGG |
| *ESR2* | Estrogen receptor 2 (all isoforms) | NM_001437 | GGCCAACACCTGGGCACCT / GGCGCAACGGTTCCCACTAAC |
| *FGFR2* | Fibroblast growth factor receptor 2 | NM_000141 | CGGTGGCTGAAAAACGGGAAG / GTACGTGTGATTGATGGACCCGTATTC |
| *FOXA1* | Forkhead box A1 | NM_004496 | CCTCCGTCCCGGTCAGCAA / GCTACTGCGCCGGGACTCAG |
| *FOXA2* | Forkhead box A2 | NM_021784 | CGCAAAGCCGCCCTACTCGT / AAGGAGAGCGAGTGGCGGATG |
| *FOXE1* | Forkhead box E1 | NM_004473 | GGGACCCTCGATGGAGCGAA / CGAGTTCCGGCAGCACATGG |
| *GATA4* | GATA Binding Protein 4 | NM_002052 | TCCTGTGCCAACTGCCAGACC / TTGGGCTTCCGTTTTCTGGTTTG |
| *GATA6* | GATA Binding Protein 6 | NM_005257 | AATAATTCCATTCCCATGACTCCAACTTC / AATACTTGAGCTCGCTGTTCTCGGG |
| *NKX2-1* | thyroid transcription factor 1 | NM_001079668 | ACGGCAACCTGGGCAACATG / GCTCACGTCCCCCAGCGAG |
| *PAX8* | Paired box 8 | NM_003466 | CCATGGCTGCGTCAGCAAGA / GGTCTCGGATCTCCCAGGCAA |
| *PCNA* | Proliferating cell nuclear antigen | NM_002592 | CGGTCTGAGGGCTTCGACACCTA / TTGGTGCTTCAAATACTAGCGCCAA |
| *SLC5A5* | Solute carrier family 5, member 5 | NM_000453.2 | CGTGCCGTCGGAGGCCTATC / GCGGCTGAAGCGCATCTCCA |
| *SOX17* | SRY (sex determining region Y)-box 17 | NM_022454 | GGGAGGTGGACCGCACGG / GCAGTAATATACCGCGGAGCTGGC |
| *TP63* | Tumor protein p63 | NM_003722 | CCGCCAAGTCGGCCACCT / CCACCTCCGTGACGTGCTCAG |
| *TPO* | Thyroid peroxidase | NM_000547 | GGCTCCCTCGGGTGACTTGG / CAGCTCGGGAGTTCCTCCGC |
| *TSHR* | Thyroid stimulating hormone receptor | M31774 | TCTTACCCAAGCCACTGCTGTGCT / CCCAGATTCTCTTCATATTCCTGGTGGA |
| ***SDHA*** | **Succinate Dehydrogenase Complex Flavoprotein Subunit A** | **NM_004168** | **ACGTCACGAAGGAGCCGATCC / ATGTACCGAGGCACAGGCGG** |

**Additional file 3: Table S2:** Gene names and primer sequences used for real-time quantitative PCR. Housekeeping genes are shown in bold.
