# Supplementary material for: Maternal smoking and high BMI disrupt thyroid gland development
Source: BMC Med. 2018 Oct 23;16:194. doi: 10.1186/s12916-018-1183-7 (PMC6198368; doi:10.1186/s12916-018-1183-7)
Supplement: Supplementary file 4 — Table S3. Significance (P values) of associations between fetal age, sex, smoke exposure, high maternal BMI, and their interactions (2-way and 3-way analyses), and thyroid weight, morphology and immunostaining. Statistically significant differences (DOCX 29 kb) [file 12916_2018_1183_MOESM4_ESM.docx]

| **Interactions:** | **3-way** | **2-way** | | | | **1-way** | | |
| --- | --- | --- | --- | --- | --- | --- | --- | --- |
| **Smoke effect** | **age-sex-SE** | ***dataset splits*** | **age-sex** | **age-SE** | **sex-SE** | **age** | **sex** | **SE** |
| weight | 0.94 |  | 0.10 | 0.55 | 0.58 | <**0.0001** (↑) | 0.22 | 0.50 |
| morphology | 0.85 |  | 0.67 | 0.52 | 0.44 | 0.69 | 0.057 | 0.75 |
| PAX8 | 0.34 |  | **0.008** | 0.29 | **0.0002** | N/A | **interaction** | **interaction** |
|  |  | *Sex split* | | | | | | |
|  |  | ♂ | N/A | 0.64 | N/A | 0.81 | N/A | 0.070 |
|  |  | **♀** | N/A | 0.14 | N/A | **0.016**(↑) | N/A | **0.003 (**↓) |
| FOXA2 | **0.042** | *Sex split* | | | | | | |
|  |  | ♂ | N/A | 0.36 | N/A | 0.55 | N/A | 0.88 |
|  |  | **♀** | N/A | 0.096 | N/A | 0.20 | N/A | 0.43 |
|  |  | *Exposure split* | | | | | | |
|  |  | C | 0.97 | N/A | N/A | 0.78 | 0.89 | N/A |
|  |  | SE | **0.011** | N/A | N/A | 0.76 | **interaction** | N/A |
| Calcitonin | 0.56 |  | 0.47 | 0.70 | 0.38 | **0.026** (↑) | 0.11 | 0.93 |
| NIS | 0.085 |  | 0.086 | 0.59 | 0.15 | **0.015** (↑) | 0.47 | 0.79 |
| **BMI effect** | **age-sex-BMI** | ***dataset splits*** | **age-sex** | **age-BMI** | **sex-BMI** | **age** | **sex** | **BMI** |
| weight | 0.64 |  | 0.26 | 0.81 | 0.80 | <**0.0001**(↑) | 0.26 | **0.046**  **(+1.18 fold)** |
| morphology | 0.80 |  | 0.88 | 0.86 | **0.034** | N/A | N/A | N/A |
|  |  | *Sex split* | | | | | | |
|  |  | ♂ | N/A | 0.77 | N/A | 0.63 | N/A | 0.89 |
|  |  | **♀** | N/A | 0.92 | N/A | 0.62 | N/A | **0.007** |
| PAX8 | 0.51 |  | 0.072 | 0.30 | 0.93 | 0.32 | 0.18 | **0.018** (↓) |
| FOXA2 | **0.005** | *Sex split* | | | | | | |
|  |  | **♂** | N/A | 0.59 | N/A | 0.47 | N/A | 0.14 |
|  |  | **♀** | N/A | **0.005** | N/A | 0.16  (BMI<25) | N/A | N/A |
|  |  | *BMI split* | | | | |  | |
|  |  | BMI<25 | 0.51 | N/A | N/A | 0.48 | 0.87 | N/A |
|  |  | BMI ≥25 | **0.0006** | N/A | N/A | 0.13 (♀) | **interaction** | N/A |
| Calcitonin | 0.96 |  | 0.50 | 0.15 | 0.29 | **0.024** (↑) | 0.44 | 0.10 |
| NIS | 0.87 |  | **0.028** | 0.32 | 0.19 | N/A | **interaction** | N/A |
|  |  | *Sex split* | | | | | | |
|  |  | ♂ | N/A | 0.44 | N/A | **0.002** (↑) | N/A | 0.31 |
|  |  | **♀** | N/A | 0.51 | N/A | 0.73 | N/A | 0.57 |

**Additional file 4: Table S3.** Significance (*P* values) of associations between fetal age, sex, smoke exposure, high maternal BMI, and their interactions (2-way and 3-way analyses), and thyroid weight, morphology and immunostaining. Statistically significant differences (*P*<0.05) are shown in bold. C: controls; SE: smoke-exposed. N/A: not applicable
