# Supplementary material for: Maternal smoking and high BMI disrupt thyroid gland development
Source: BMC Med. 2018 Oct 23;16:194. doi: 10.1186/s12916-018-1183-7 (PMC6198368; doi:10.1186/s12916-018-1183-7)
Supplement: Supplementary file 5 — Table S4. Significance (P values) of associations between fetal age, sex, smoke exposure, and their interactions (2-way and 3-way analyses), and normalized fetal thyroidal transcripts. Data were log-transformed for those cases where model residuals departed from normality. Statistically significant differences (P < 0.05) are shown in bold. C: controls; SE: smoke-exposed. N/A, not applicable. (DOCX 30 kb) [file 12916_2018_1183_MOESM5_ESM.docx]

| **Interaction:** | **3-way** |  | **2-way** | | | **1-way** | | |
| --- | --- | --- | --- | --- | --- | --- | --- | --- |
|  | **age-sex-SE** | ***dataset splits*** | **age-sex** | **age-SE** | **sex-SE** | **age** | **sex** | **SE** |
| *AHR* | 0.9 |  | 0.46 | **0.021** | 0.87 | N/A | N/A | **interaction** |
|  |  | *Exposure split* | | | | | | |
|  |  | C | 0.53 | N/A | N/A | **0.007** (↑) | **0.014**  **(-1.3 fold ♂)** | N/A |
|  |  | SE | 0.67 | N/A | N/A | 0.82 | **0.039**  **(-1.3 fold ♂)** | N/A |
| *AR* | 0.35 |  | 0.68 | 0.71 | 0.94 | 0.59 | **0.004**  **(-1.4 fold ♂)** | 0.88 |
| *ARNT* | 0.76 |  | 0.3 | 0.13 | 0.55 | 0.56 | 0.06  (-1.2 fold ♂) | 0.78 |
| *BAX-BCL2* ratio | 0.74 |  | **0.038** | 0.99 | 0.87 | N/A | **interaction** | N/A |
|  |  | *Sex split* | | | | | | |
|  |  | ♂ | N/A | 0.82 | N/A | **0.014** (↑) | N/A | 0.26 |
|  |  | **♀** | N/A | 0.81 | N/A | 0.46 | N/A | 0.26 |
| *BAX* | 0.63 |  | 0.088 | 0.49 | 0.91 | **0.01** (↑) | 0.53 | 0.085 |
| *BCL2* | 0.75 |  | 0.62 | 0.25 | 0.76 | **0.003** (↑) | 0.1 | 0.81 |
| *ESR1* | 0.96 |  | 0.54 | 0.23 | 0.83 | **0.0001** (↑) | 0.66 | 0.22 |
| *ESR2* | 0.24 |  | 0.69 | **0.018** | 0.62 | N/A | N/A | **interaction** |
|  |  | *Exposure split* | | | | | | |
|  |  | C | 0.68 | N/A | N/A | 0.34 | 0.76 | N/A |
|  |  | SE | 0.22 | N/A | N/A | **0.026** (↓) | 0.38 | N/A |
| *FGFR2* | 0.75 |  | 0.84 | 0.6 | 0.23 | 0.13 | 0.2 | 0.99 |
| *FOXA1* | 0.61 |  | 0.36 | 0.23 | 0.51 | 0.29 | 0.73 | 0.46 |
| *FOXA1-FOXA2* ratio | 0.66 |  | 0.72 | 0.87 | 0.54 | 0.72 | 0.32 | 0.57 |
| *FOXA2* | 0.15 |  | 0.18 | 0.89 | 0.27 | **0.025** (↑) | 0.39 | 0.86 |
| *FOXE1* | 0.31 |  | 0.97 | 0.44 | 0.17 | 0.58 | 0.81 | 0.17 |
| *GATA4* | 0.4 |  | 0.37 | 0.33 | 0.86 | **0.024** (↓) | 0.88 | 0.59 |
| *GATA6* | 0.75 |  | 0.12 | 0.12 | 0.96 | 0.76 | 0.60 | **0.021**  **(-1.7 fold)** |
| *NKX2-1* | 0.76 |  | 0.86 | 0.81 | 0.26 | 0.81 | 0.88 | **0.006**  **(-1.4 fold)** |
| *PAX8* | 0.24 |  | 0.16 | 0.12 | 0.4 | 0.14 | 0.24 | 0.59 |
| *PCNA* | 0.36 |  | 0.65 | 0.24 | 0.63 | 0.33 | 0.87 | 0.27 |
| *SLC5A5* | 0.62 |  | 0.56 | 0.99 | 0.71 | 0.28 | 0.85 | 0.63 |
| *SOX17* | **0.043** | *Sex split* | | | | | | |
|  |  | ♂ | N/A | 0.19 | N/A | 0.28 | N/A | 0.20 |
|  |  | **♀** | N/A | 0.12 | N/A | 0.58 | N/A | 0.10 |
|  |  | *Exposure split* | | | | | | |
|  |  | C | 0.22 | N/A | N/A | 0.57 | 0.74 | N/A |
|  |  | SE | 0.11 | N/A | N/A | 0.15 | 0.40 | N/A |
| *TP63* | 0.79 |  | 0.85 | 0.58 | 0.79 | 0.66 | 0.67 | 0.42 |
| *TPO* | 0.84 |  | 0.52 | 0.93 | 0.66 | **0.009** (↑) | 0.49 | 0.9 |
| *TSHR* | 0.95 |  | 0.92 | 0.12 | 0.33 | 0.77 | 0.22 | **0.046**  **(-1.3 fold)** |

**Additional file 5: Table S4.** Significance (*P* values) of associations between fetal age, sex, smoke exposure, and their interactions (2-way and 3-way analyses), and normalised fetal thyroidal transcripts. Data were log-transformed for those cases where model residuals departed from normality. Statistically significant differences (*P*<0.05) are shown in bold. C: controls; SE: smoke-exposed. N/A, not applicable.
