# Supplementary material for: Maternal smoking and high BMI disrupt thyroid gland development
Source: BMC Med. 2018 Oct 23;16:194. doi: 10.1186/s12916-018-1183-7 (PMC6198368; doi:10.1186/s12916-018-1183-7)

Supplementary Figure 2

A. Sex differences in concentrations of fetal plasma hormones

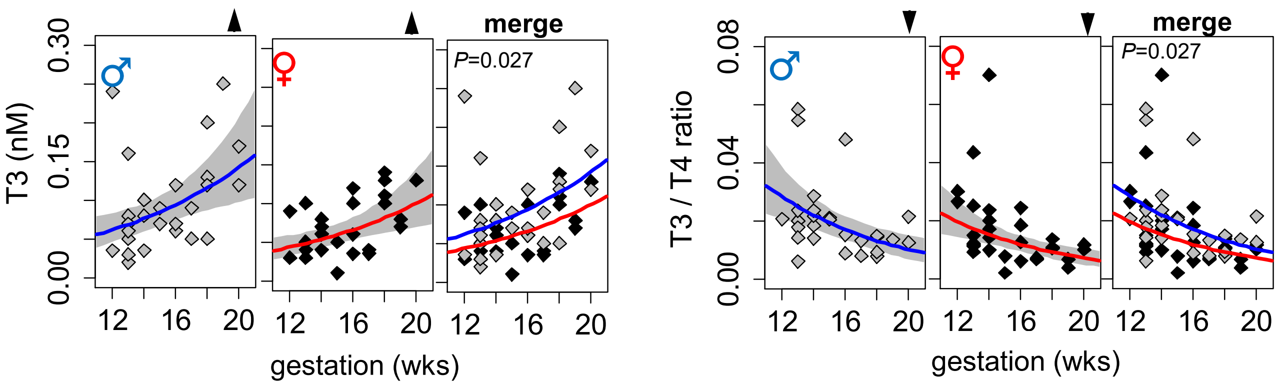

B. Sex differences in fetal thyroid immunohistochemical staining scores

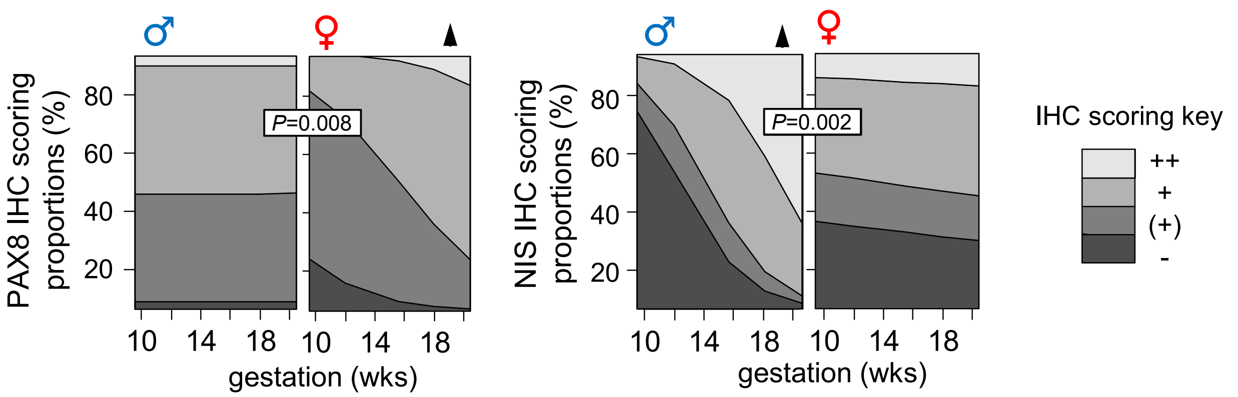

C. Sex differences in fetal thyroid transcript expression levels

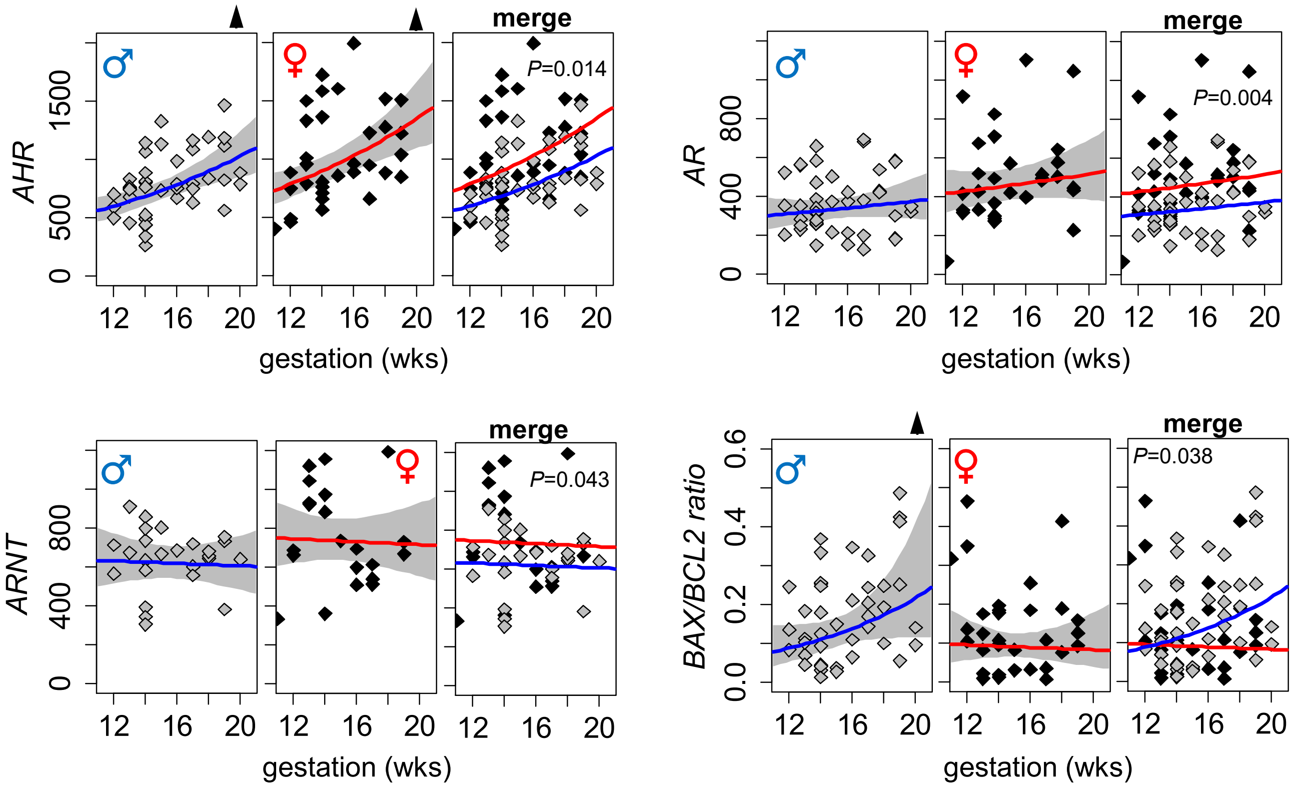

Supplement: Supplementary file 6 — Figure S2. Statistically significant (P < 0.05) effects of fetal sex on A. circulating hormones, B. immunohistochemistry scores, and C. transcripts. Shaded areas in A and C represent 95% confidence intervals. Immunohistochemistry scoring proportions in B are shown as stacked percentages for each scoring (“−, unstained” < “(+)” < “+” < “++, more stained”) across gestation. Arrowheads indicate significant (P < 0.05) increase (▲) or decrease (▼) by gestational age. P values associated with changes in relation to smoke exposure are provided in the merged graph panels. (PDF 474 kb) [file 12916_2018_1183_MOESM6_ESM.pdf]
