# Supplementary material for: Maternal smoking and high BMI disrupt thyroid gland development
Source: BMC Med. 2018 Oct 23;16:194. doi: 10.1186/s12916-018-1183-7 (PMC6198368; doi:10.1186/s12916-018-1183-7)
Supplement: Supplementary file 7 — Table S5. Significance (P values) of associations between fetal age, sex, maternal BMI, and their interactions (2-way and 3-way analyses), and normalized human fetal thyroid transcripts. Data were log-transformed for those cases where model residuals departed from normality. Statistically significant differences (P < 0.05) are shown in bold. N/A: not applicable. (DOCX 32 kb) [file 12916_2018_1183_MOESM7_ESM.docx]

**Additional file 7: Table S5.** Significance (*P* values) of associations between fetal age, sex, maternal BMI, and their interactions (2-way and 3-way analyses), and normalised human fetal thyroid transcripts. Data were log-transformed for those cases where model residuals departed from normality. Statistically significant differences (*P*<0.05) are shown in bold. N/A: not applicable.

| **Interaction:** | **3-way** |  | **2-way** | | | **1-way** | | |
| --- | --- | --- | --- | --- | --- | --- | --- | --- |
|  | **age-sex-BMI** | ***dataset splits*** | **age-sex** | **age-BMI** | **sex-BMI** | **age** | **sex** | **BMI** |
| *AHR* | 0.97 |  | 0.64 | **0.018** | 0.18 | N/A | N/A | **interaction** |
|  |  | *BMI split* | | | | | | |
|  |  | BMI<25 | 0.70 | N/A | N/A | **<0.0001** (↑) | 0.063  **(-1.2 fold ♂)** | N/A |
|  |  | BMI≥25 | 0.77 | N/A | N/A | 0.77 | **0.0014**  **(-1.5 fold ♂)** | N/A |
| *AR* | 0.97 |  | 0.92 | 0.32 | 0.08 | 0.65 | **0.002**  **(-1.4 fold ♂)** | 0.14 |
| *ARNT* | 0.68 |  | 0.43 | 0.73 | 0.09 | 0.48 | **0.043**  **(-1.2 fold ♂)** | 0.54 |
| *BAX-BCL2* ratio | 0.49 |  | **0.045** | 0.15 | 0.91 | 0.42 | 0.44 | 0.84 |
|  |  | *Sex split* | | | | | | |
|  |  | **♂** | N/A | 0.52 | N/A | **0.02** (↑) | N/A | 0.96 |
|  |  | ♀ | N/A | 0.15 | N/A | 0.123 | N/A | 0.83 |
| *BAX* | 0.065 |  | 0.15 | 0.28 | 0.96 | **0.02** (↑) | 0.82 | 0.77 |
| *BCL2* | 0.31 |  | 0.32 | 0.75 | 0.38 | **0.003** (↑) | 0.07 | 0.57 |
| *ESR1* | 0.39 |  | 0.36 | **0.002** | 0.11 | N/A | N/A | **interaction** |
|  |  | *BMI split* | | | | | | |
|  |  | BMI<25 | 0.28 | N/A | N/A | **0.005** (↑) | 0.66 | N/A |
|  |  | BMI≥25 | 0.97 | N/A | N/A | **0.041** (↑) | **0.033**  **(-1.4 fold ♂)** | N/A |
| *ESR2* | 0.81 |  | 0.55 | 0.09 | 0.16 | 0.38 | 0.37 | 0.40 |
| *FGFR2* | 0.74 |  | 0.7 | 0.36 | 0.25 | 0.14 | 0.19 | 0.71 |
| *FOXA1* | 0.15 |  | 0.6 | 0.65 | 0.54 | 0.23 | 0.92 | 0.37 |
| *FOXA1-FOXA2* ratio | **0.028** | *Sex split* | | | | | | |
|  |  | **♂** | N/A | 0.11 | N/A | 0.097 (↑) | N/A | 0.95 |
|  |  | ♀ | N/A | 0.14 | N/A | 0.082 (↓) | N/A | 0.064 |
|  |  | *BMI split* | | | | | | |
|  |  | BMI<25 | **0.024** | N/A | N/A | 0.080 (↓♀) | **interaction** | N/A |
|  |  | BMI≥25 | 0.41 | N/A | N/A | 0.87 | 0.46 | N/A |
| *FOXA2* | 0.25 |  | 0.27 | 0.79 | 0.21 | **0.027** (↑) | 0.37 | 0.48 |
| *FOXE1* | **0.021** | *Sex split* | | | | | | |
|  |  | **♂** | N/A | 0.14 | N/A | 0.23 | N/A | 0.21 |
|  |  | ♀ | N/A | **0.041** | N/A | 0.059 (↑BMI<25) | N/A | **interaction** |
|  |  | *BMI split* | | | | | | |
|  |  | BMI<25 | 0.056 | N/A | N/A | 0.064(↑♀) | 0.45 | N/A |
|  |  | BMI≥25 | 0.16 | N/A | N/A | 0.82 | 0.78 | N/A |
| *GATA4* | 0.38 |  | 0.53 | 0.81 | 0.53 | **0.02** (↓) | 0.79 | 0.91 |
| *GATA6* | 0.97 |  | 0.2 | 0.33 | 0.54 | 0.57 | 0.15 | 0.56 |
| *NKX2.1* | 0.55 |  | 0.91 | 0.83 | 0.27 | 0.64 | 0.7 | 0.48 |
| *PAX8* | 0.3 |  | 0.39 | 0.83 | 0.53 | 0.19 | 0.15 | 0.14 |
| *PCNA* | 0.57 |  | 0.39 | 0.85 | 0.87 | 0.37 | 0.97 | 0.56 |
| *SLC5A5* | 0.69 |  | 0.44 | 0.26 | 0.84 | 0.28 | 0.85 | 0.63 |
| *SOX17* | 0.23 |  | 0.85 | 0.53 | 0.26 | 0.64 | 0.69 | 0.68 |
| *TP63* | 0.56 |  | 0.61 | 0.74 | 0.29 | 0.54 | 0.5 | 0.33 |
| *TPO* | 0.99 |  | 0.40 | 0.20 | 0.86 | **0.009** (↑) | 0.47 | 0.86 |
| *TSHR* | 0.30 |  | 0.49 | 0.61 | 0.47 | 0.50 | 0.48 | 0.98 |
