# Supplementary material for: Maternal smoking and high BMI disrupt thyroid gland development
Source: BMC Med. 2018 Oct 23;16:194. doi: 10.1186/s12916-018-1183-7 (PMC6198368; doi:10.1186/s12916-018-1183-7)

Supplementary Figure 3

A. Sex-specific correlations between fetal levels of TSH, cotinine and T4

i. TSH and cotinine correlations *ii.* TSH and T4 correlations

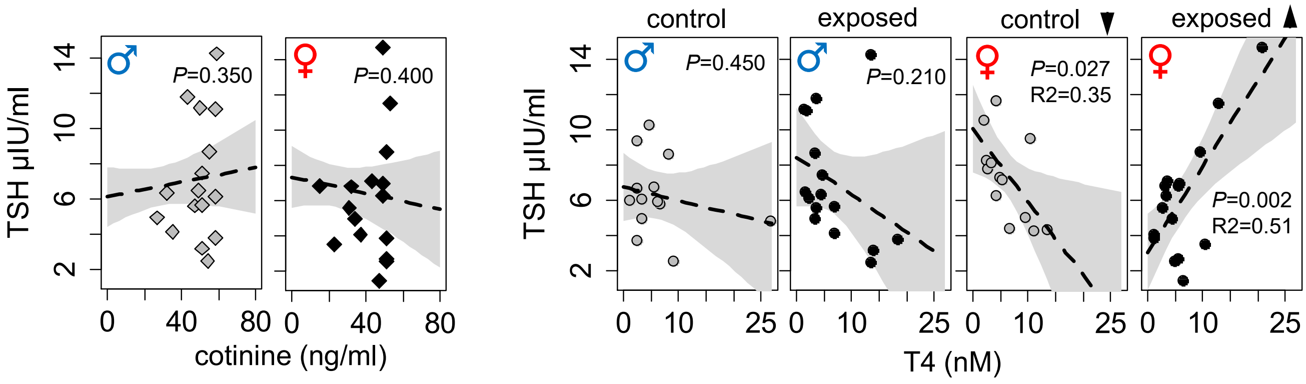

B. Sex-specific correlation between maternal BMI and circulating fetal TSH levels

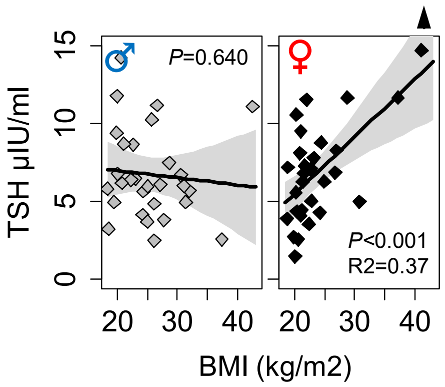

C. Associations between maternal smoking, maternal BMI and fetal TSH levels in female fetuses

i. Control (non-smoke exposed) females

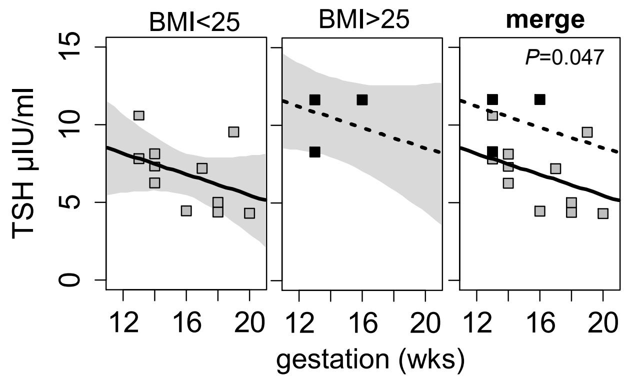

ii. Smoke exposed females

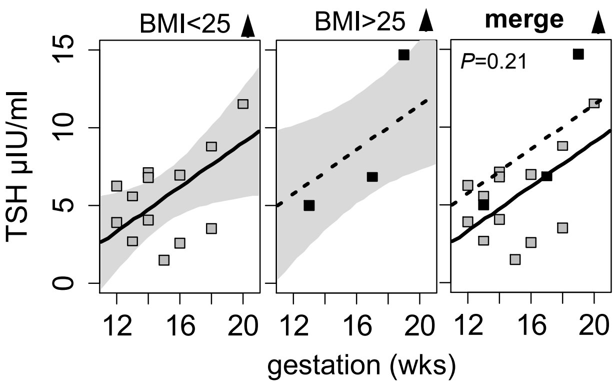

iii. Correlation between TSH and maternal BMI in control (non-smoke exposed) females

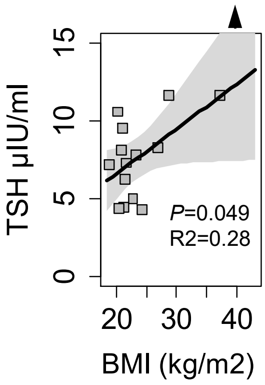

iv. Correlation between TSH and maternal BMI in smoke exposed female fetuses

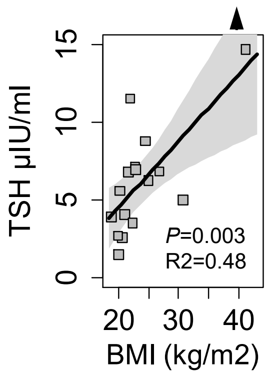

Supplement: Supplementary file 8 — Figure S3. A. Correlations among TSH, cotinine, and T4 in the human fetal circulation. B. Correlation between fetal TSH and maternal BMI (continuous). C. Associations between smoking, maternal BMI, and fetal TSH levels in the female fetuses. In Ci-ii, the females were stratified according to smoke exposure and the effect of age and BMI (categorical) on TSH was examined. In Ciii-iv, TSH levels were correlated to BMI (continuous) in the control and smoke-exposed females. Pearson’s correlation P values in A, B, Ciii, and Civ are given within the graphs, including R2 values in the cases of statistically significance (P < 0.05). Arrowheads above the graphs indicate significant (P < 0.05) increase (▲) or decrease (▼). (PDF 451 kb) [file 12916_2018_1183_MOESM8_ESM.pdf]
